# The replication-competent HIV reservoir is a genetically restricted, younger subset of the overall pool of HIV proviruses persisting during therapy, which is highly genetically stable over time

**DOI:** 10.1128/jvi.01655-23

**Published:** 2024-01-12

**Authors:** Aniqa Shahid, Signe MacLennan, Bradley R. Jones, Hanwei Sudderuddin, Zhong Dang, Kyle Cobarrubias, Maggie C. Duncan, Natalie N. Kinloch, Michael J. Dapp, Nancie M. Archin, Margaret A. Fischl, Igho Ofotokun, Adaora Adimora, Stephen Gange, Bradley Aouizerat, Mark H. Kuniholm, Seble Kassaye, James I. Mullins, Harris Goldstein, Jeffrey B. Joy, Kathryn Anastos, Zabrina L. Brumme, the MACS/WIHS combined cohort study (MWCSS), Ighovwerha Ofotokun

**Affiliations:** 1 Emory University, Atlanta, Georgia, USA; 2 Johns Hopkins University, Baltimore, Maryland, USA; 3 Albert Einstein College of Medicine, Bronx, New York, USA; 4 Suny Downstate Medical Center, Brooklyn, New York, USA; 5 Johns Hopkins University, Baltimore, Maryland, USA; 6 Hektoen Institute for Medical Research, Chicago, Illinois, USA; 7 Northwestern University at Chicago, Chicago, Illinois, USA; 8 University of California San Francisco, San Francisco, California, USA; 9 University of California Los Angeles, Los Angeles, California, USA; 10 Georgetown University, Washington, DC, USA; 11 University of Miami School of Medicine, Coral Gables, Florida, USA; 12 University of Pittsburgh, Pittsburgh, Pennsylvania, USA; 13 University of Alabama Birmingham, Birmingham, Alabama, USA; 14 University of North Carolina Chapel Hill, Chapel Hill, North Carolina, USA; 1 Faculty of Health Sciences, Simon Fraser University, Burnaby, British Columbia, Canada; 2 British Columbia Centre for Excellence in HIV/AIDS, Vancouver, British Columbia, Canada; 3 Bioinformatics Program, University of British Columbia, Vancouver, British Columbia, Canada; 4 Department of Microbiology, University of Washington, School of Medicine, Seattle, Washington, USA; 5 UNC HIV Cure Center, Institute of Global Health and Infectious Diseases, University of North Carolina at Chapel Hill, Chapel Hill, North Carolina, USA; 6 Department of Medicine, University of Miami School of Medicine, Miami, Florida, USA; 7 Division of Infectious Diseases, Department of Medicine, Emory University School of Medicine, Atlanta, Georgia, USA; 8 Department of Epidemiology, UNC Gillings School of Global Public Health, University of North Carolina at Chapel Hill, Chapel Hill, North Carolina, USA; 9 Department of Epidemiology, Johns Hopkins Bloomberg School of Public Health, Baltimore, Maryland, USA; 10 College of Dentistry, New York University, New York, New York, USA; 11 Department of Epidemiology and Biostatistics, University at Albany, State University of New York, Rensselaer, New York, New York, USA; 12 Division of Infectious Diseases and Tropical Medicine, Georgetown University, Washington, DC, USA; 13 Department of Global Health, University of Washington, School of Medicine, Seattle, Washington, USA; 14 Department of Medicine, University of Washington, School of Medicine, Seattle, Washington, USA; 15 Departments of Microbiology and Immunology and Pediatrics, Albert Einstein College of Medicine, Bronx, New York, New York, USA; 16 Department of Medicine, University of British Columbia, Vancouver, British Columbia, Canada; 17 Department of Medicine, Albert Einstein College of Medicine, New York, New York, USA; Ulm University Medical Center, Ulm, Germany

**Keywords:** HIV, persistence, genetic stability, molecular dating, phylogenetics, rebound

## Abstract

Within-host HIV populations continually diversify during untreated infection, and this diversity persists within infected cell reservoirs during antiretroviral therapy (ART). Achieving a better understanding of on-ART proviral evolutionary dynamics, and a better appreciation of how the overall persisting pool of (largely genetically defective) proviruses differs from the much smaller replication-competent HIV reservoir, is critical to HIV cure efforts. We reconstructed within-host HIV evolutionary histories in blood from seven participants of the Women’s Interagency HIV Study who experienced HIV seroconversion, and used these data to characterize the diversity, lineage origins, and ages of proviral *env-gp120* sequences sampled longitudinally up to 12 years on ART. We also studied HIV sequences emerging from the reservoir in two participants. We observed that proviral clonality generally increased over time on ART, with clones frequently persisting long term. While on-ART proviral integration dates generally spanned the duration of untreated infection, HIV emerging in plasma was exclusively younger (i.e., dated to the years immediately pre-ART). The genetic and age distributions of distinct proviral sequences remained stable during ART in all but one participant, in whom there was evidence that younger proviruses had been preferentially eliminated after 12 years on ART. Analysis of the *gag* region in three participants corroborated our *env-gp120*-based observations, indicating that our observations are not influenced by the HIV region studied. Our results underscore the remarkable genetic stability of the distinct proviral sequences that persist in blood during ART. Our results also suggest that the replication-competent HIV reservoir is a genetically restricted, younger subset of this overall proviral pool.

**IMPORTANCE:**

Characterizing the genetically diverse HIV sequences that persist in the reservoir despite antiretroviral therapy (ART) is critical to cure efforts. Our observations confirm that proviruses persisting in blood on ART, which are largely genetically defective, broadly reflect the extent of within-host HIV evolution pre-ART. Moreover, on-ART clonal expansion is not appreciably accompanied by the loss of distinct proviral lineages. In fact, on-ART proviral genetic composition remained stable in all but one participant, in whom, after 12 years on ART, proviruses dating to around near ART initiation had been preferentially eliminated. We also identified recombinant proviruses between parental sequence fragments of different ages. Though rare, such sequences suggest that reservoir cells can be superinfected with HIV from another infection era. Overall, our finding that the replication-competent reservoir in blood is a genetically restricted, younger subset of all persisting proviruses suggests that HIV cure strategies will need to eliminate a reservoir that differs in key respects from the overall proviral pool.

## INTRODUCTION

The ability of HIV to persist as an integrated provirus within a small fraction of infected cells, even during suppressive antiretroviral therapy (ART), is the main barrier to cure (1, 2). It is also the reason why ART must be taken for life. Seeding of HIV sequences into reservoir cells begins immediately following infection (3, 4) and continues until viral suppression is achieved on ART, thereby establishing a genetically diverse viral reservoir (5–9). Understanding the within-host evolutionary dynamics of the proviruses that persist during ART, as well as the origins of HIV sequences that emerge from the reservoir if ART is interrupted, will aid the development of curative strategies.

In recent years, our understanding of reservoir dynamics has been enriched by studies that have interpreted on-ART proviral genetic diversity in blood in the context of HIV’s within-host evolutionary history (5–7, 10–12). These studies have revealed that a large percentage of proviruses that persist in the blood during ART (most of which are genetically defective (13–15)), as well as the vast majority of replication-competent reservoir sequences that persist during this time, date to the year or two preceding ART initiation (5, 6, 9–11). We now understand that this is because reservoir turnover during untreated infection is relatively rapid (the half-life of persisting proviruses during this period is estimated to be a year or less (5, 16)). This means that, if ART is not initiated until advanced chronic infection, many of the earliest within-host lineages will have already been eliminated by this time. Nevertheless, proviruses dating to earlier periods of infection are routinely recovered during ART, albeit less frequently (5–7, 9, 11).

During the initial years of ART, the proviral pool decreases in size (initial on-ART half-lives of intact and defective proviruses are ~4 and >10 years, respectively, with decay slowing further thereafter (17–19)). At the same time, clonal expansion of infected cells also occurs (20–22). Given these opposing processes, and assuming that no new viral variants are seeded into the reservoir during ART (5, 23), it is reasonable to hypothesize that the persisting proviral pool will decline in genetic diversity over time, as distinct proviruses are gradually eliminated. Relatively few studies, however, have investigated on-ART proviral genetic stability (20, 24–29). Moreover, only two have done so in the context of HIV’s within-host evolutionary history (5, 6), which can shed light on the lineage origins and ages of persisting proviruses. Their results however were not entirely concordant. While one study suggested that younger HIV lineages may be preferentially eliminated during the initial years of ART (though this did not reach statistical significance (6)), the other supported relative proviral genetic stability even in the longer term (though the primary goal of the latter analysis was to investigate whether residual HIV replication occurs during ART, not to evaluate proviral genetic stability over time (5)). Even fewer studies have compared the within-host evolutionary origins and ages of proviruses persisting on ART with those of HIV sequences emerging from the reservoir (i.e., as rebound viremia) (30), which have been shown to include within-host recombinants of unknown origin (31). Such analyses can help illuminate how the rebound-competent reservoir in blood may be distinctive from the overall, largely defective, proviral pool.

To address these knowledge gaps, we reconstructed within-host HIV evolutionary histories in seven participants enrolled in the Women’s Interagency HIV Study (WIHS) who seroconverted during follow-up. Our goal was to investigate the genetic stability of distinct proviruses sampled up to four times, up to 12 years following ART initiation. In two participants, we also investigated the diversity and age distribution of reservoir-origin HIV sequences that emerged in plasma post-ART.

## RESULTS

### Participant characteristics and sampling

**Fig 1:**
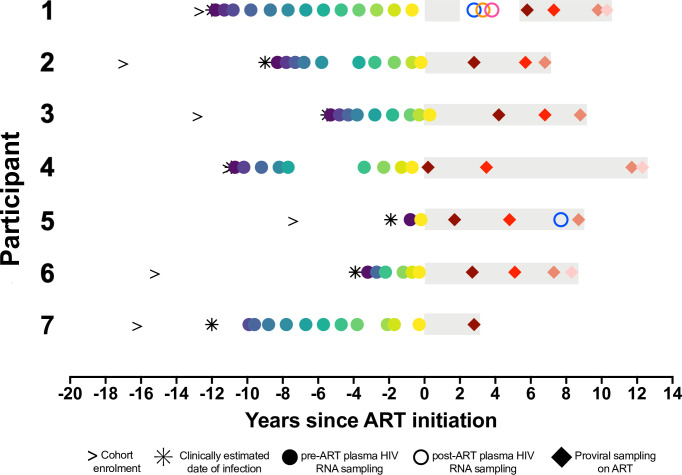
Participant sampling timeline. Time zero denotes ART initiation. The right arrow denotes enrolment into the cohort. The asterisk denotes the clinically estimated date of infection, defined as the midpoint between the last negative and first positive HIV tests. Gray shading denotes ART. Here and throughout all figures, closed circles denote pre-ART plasma HIV RNA sampling. Open circles denote post-ART plasma HIV RNA sampling. Diamonds denote proviral sampling on ART. Participants 1, 3, and 7 correspond to participants 1, 3, and 6 in reference (32).

We reconstructed the within-host HIV evolutionary histories of seven women who initiated ART at a median of 9 (range 1.9–12) years following their estimated infection dates, which were calculated as the midpoint between their last negative and first seropositive HIV visits (Fig. 1; Table 1). We leveraged this information to characterize the genetics and dynamics of proviruses sampled longitudinally during ART, as well as HIV RNA sequences rebounding in plasma. Together, we analyzed 1,092 single-genome-amplified intact HIV RNA *env-gp120* sequences (median 181, range 50–239 per participant) from a median of 9 (range 2–13) plasma samples collected over a median of 8.3 (range 0.8–11.8) years pre-ART, where these sequences were previously published for three participants (32) (Fig. 1). In addition, we analyzed 926 intact proviral *env-gp120* sequences (median 150, range 42–182 per participant) from a median of 3 time points (range 1–4) spanning a median of 8.7 (range 2.8–12.3) years during ART. For participant 1, we also analyzed 114 plasma HIV RNA *env-gp120* sequences isolated during a viremia rebound event, and for participant 5, we also analyzed four plasma HIV RNA *env-gp120* sequences isolated during initial loss of viral control. All participants had HIV subtype B, with no evidence of dual or super-infection (Fig. S1). As expected (8, 32–34), the overall extent of within-host HIV diversity correlated strongly with the duration of untreated infection (Spearman’s ρ = 0.85, *P* = 0.03; Fig. S1, *inset*).

**TABLE 1:** Participant information, HIV sampling, and sequencing details

ID	Estimated date of infection	Duration of uncontrolled infection (years)	No. of pre-ART plasma HIV RNA time points	Pre-ART plasma HIV *env-gp120* sequences Total *N* (distinct *N*; %)	ART initiation date	No. of post-ART plasma HIV RNA time points^*b*^	Post-ART plasma HIV RNA *env-gp120* sequences Total *N* (distinct *N*; %)	Years of ART until last proviral sampling	No. of on-ART proviral time points	On-ART *env-gp120* proviral sequences Total *N* (distinct *N*; %)
1	December 1995	12	13	207 (207; 100%)	January 2008	3	114 (97; 85%)	10.3	4	171 (114; 67%)
2	January 2003	9	10	239 (227; 95%)	January 2012	–	–	6.8	3	95 (79; 83%)
3	July 2002	5.5	9	140 (132; 94%)	January 2008	–	–	8.8	3	150 (63; 42%)
4^*a*^	July 1995	10.9	9	195 (182; 93%)	June 2006	–	–	12.3	4	182 (165; 91%)
5	March 2008	1.9	2	50 (45; 90%)	February 2010	1	4 (2; 50%)	8.7	3	110 (71; 65%)
6	August 2006	3.9	6	80 (73; 91%)	July 2010	–	–	8.3	4	176 (84; 48%)
7	September 1999	11.9	11	181 (181; 100%)	August 2011	–	–	2.8	1	42 (25; 60%)

^*a*^ Clinical records indicated that participant 4 initiated ART in 2003, but no reductions in plasma viral load (pVL) were observed until June 2006. For this reason, we considered June 2006 as this participant’s effective ART start date.

^*b*^ Participant 1 experienced a prolonged plasma rebound event, while participant 5 experienced an initial loss of viral control on ART, as shown in Figures 3A and 8A, respectively.

^*c*^ –, the specified sequence type was not collected for that participant.

### Proviral clonal dynamics during the initial years of ART

**Fig 2:**
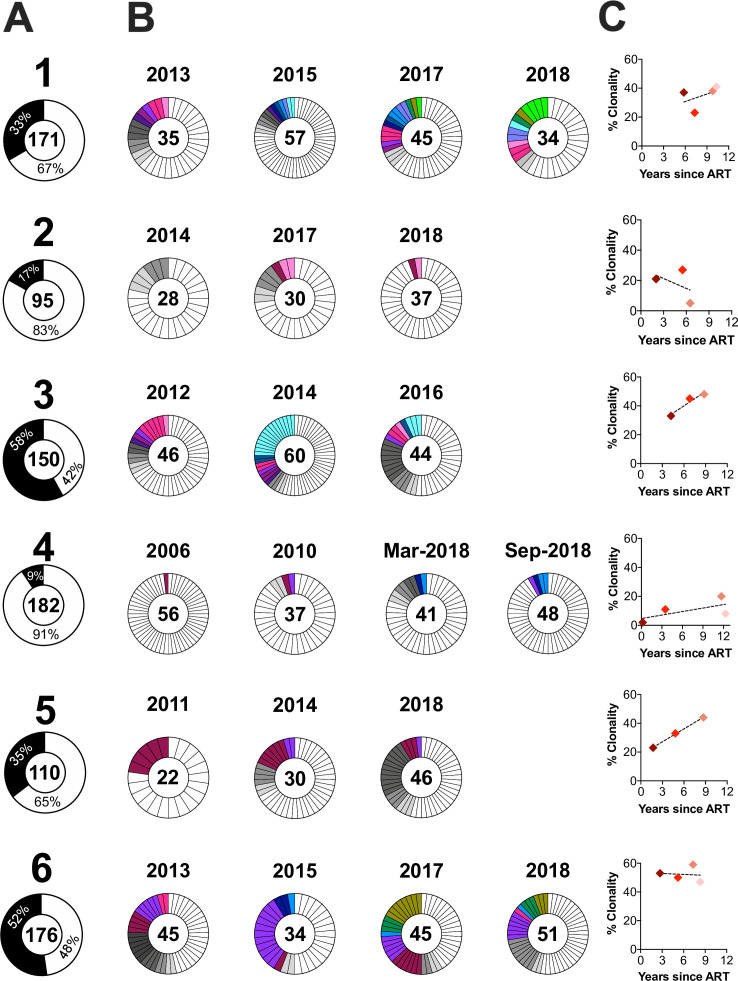
Proviral clonal distribution and dynamics during ART. (**A**) Total number of *env-gp120* proviral sequences collected per participant (shown inside the donut), and the percentages that were observed only once (white) versus those observed more than once (i.e., clones; black). (**B**) Proviral clonality by time point on ART. Gray slices denote clones observed only at that time point (each clone in a distinct shade of gray); colored slices link clones isolated across time points. (**C**) Percent proviral clonality over time on ART, with regression line.

For participants 1 through 6, we sampled proviruses at a minimum of three time points during the initial years of ART, allowing us to investigate clonal dynamics. The overall percentage of putatively clonal sequences, defined as those that matched at least one other sequence with 100% nucleotide identity in *env-gp120*, ranged from 9% (participant 4) to 58% (participant 3) (Fig. 2A). In all participants, we recovered clones that were observed at only one on-ART time point (gray slices, Fig. 2B) and across more than one time point (colored slices, Fig. 2B). In participants 3, 5, and 6, we recovered at least one clone that persisted across all on-ART time points. The proportion of clonal proviruses increased or remained stable over time in five of six participants (Fig. 2C).

### Proviral ages, within-host origins, and dynamics during ART

We used a phylogenetic approach (7) to investigate the ages, within-host evolutionary origins, and dynamics of proviral lineages persisting during ART. Only intact, non-hypermutated sequences that showed no evidence of within-host recombination were included in this analysis (recombinants were analyzed separately; see below). To mitigate the inherent uncertainty in within-host HIV evolutionary reconstruction and to allow us to estimate error in the parameters of interest (e.g., proviral integration dates; population genetic structure), we inferred a minimum 1,500 trees per participant and conditioned results across all trees. We rooted each tree at the location that maximized the correlation between the root-to-tip distances of the pre-ART plasma HIV RNA sequences and their sampling dates (as within-host sequence divergence from the transmitted/founder virus increases over time during untreated infection (32, 34, 35)). This root location represents the most recent common ancestor of the data set, which should be the transmitted/founder virus (or a close descendant) in this cohort of seroconverters. We then fit a linear model to each tree relating the root-to-tip genetic distances of distinct pre-ART plasma *env-gp120* sequences to their sampling dates. Here, the slope represents the within-host pre-ART *env-gp120* evolutionary rate, and the x-intercept represents the phylogenetically estimated infection date. This linear model was then used to convert the root-to-tip distance of each post-ART sequence of interest to its integration date. Each sequence’s estimated integration date was then averaged over all within-host trees that passed quality control (QC) (see Materials and Methods) and reported along with its 95% highest posterior density (HPD) interval.

### Participant 1

**Fig 3:**
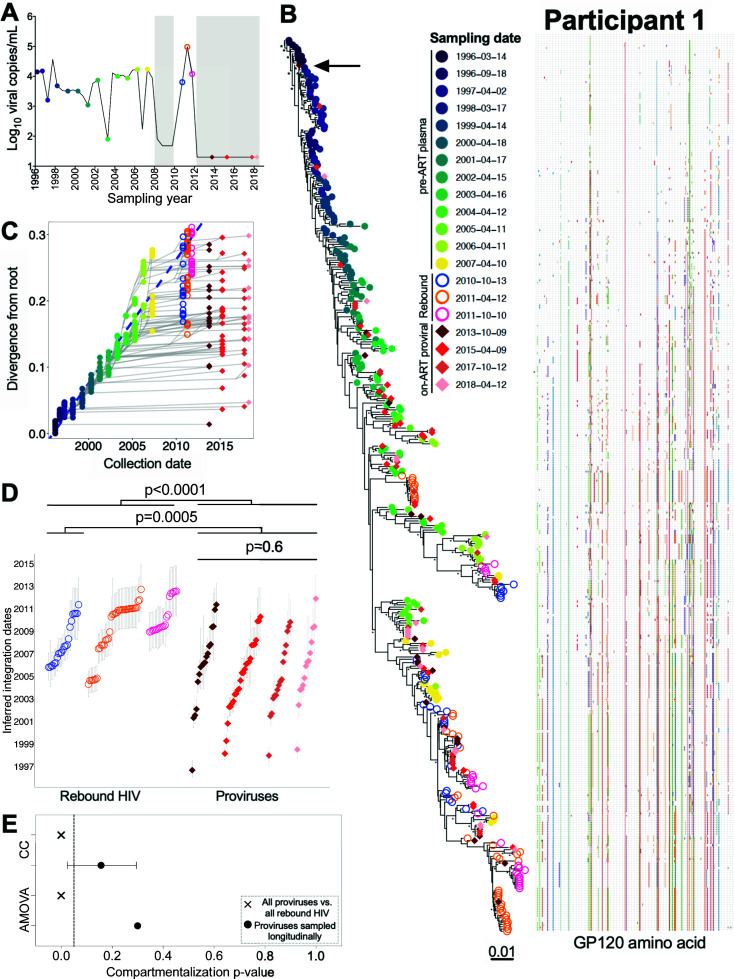
Participant 1: diversity and inferred integration dates of HIV sequences persisting during ART. (**A**) Plasma viral load history, with symbols denoting sampling time points. The lower limit of quantification of the viral load assays performed between 2008 and 2010 was 80 or 48 HIV RNA copies/mL depending on the assay; thereafter, it was 20 copies HIV RNA copies/mL. Closed circles denote pre-ART plasma HIV RNA sampling, and open circles denote post-ART plasma HIV RNA sampling. Diamonds denote proviral sampling on ART. Gray shading denotes ART. (**B**) Example rooted within-host phylogeny, with scale in estimated substitutions per nucleotide site. Asterisks identify nodes supported by posterior probabilities ≥ 70%. The adjacent highlighter plot shows ENV-GP120 amino acid diversity, with colored ticks denoting non-synonymous substitutions with respect to the reference sequence at the top of the phylogeny. (**C**) Linear model (dashed blue diagonal) relating plasma HIV RNA collection dates (closed colored circles) to their respective root-to-tip distances in the example phylogeny shown in **B**. This linear model is then used to convert the root-to-tip distances of on-ART sequences of interest (rebound HIV in open circles and proviruses in colored diamonds) to their integration dates. Gray lines trace the phylogenetic relationships between HIV *env-gp120* sequences. (**D**) Integration dates and 95% HPD intervals for distinct on-ART sequences, stratified by collection year, that were derived from averaging results across all QC-passed phylogenies for this participant. The Kruskal-Wallis test was used to compare proviral integration date distributions across all sampling time points. The Mann-Whitney U-test was used to compare plasma and proviral integration date distributions. (**E**) This panel shows the *P*-values from the tests for population genetic structure (AMOVA, = analysis of molecular variance; CC, correlation coefficient) that compared the populations of distinct proviral sequences per time point (closed black circle) and the distinct proviral and rebound populations overall (cross symbol). The bars around the CC *P*-value represent the 95% HPD interval of the *P*-values derived from all QC-passed phylogenies for this participant.

Participant 1 was estimated to have acquired HIV in late 1995 but only initiated ART in January 2008 (Fig. 1 and 3A). We inferred 15,000 within-host phylogenies relating 207 plasma HIV RNA *env-gp120* sequences collected over 12 years of untreated infection (these sequences were originally published in (32)), 97 plasma HIV RNA *env-gp120* sequences collected at three time points during a viremia rebound event, and 90 proviral *env-gp120* sequences collected at four subsequent time points after viremia was re-suppressed on ART (Table 1; Table S1). Of these trees, 7,218 passed QC (see Materials and Methods and Table S1). The trees exhibited the ladder-like shape typical of within-host HIV evolution, where plasma HIV sequences sampled during untreated infection were increasingly divergent from the root over time (example phylogeny in Fig. 3B). This shape is the result of serial genetic bottlenecks imposed by immune responses from which the virus continually escapes; the selective sweeps that characterize this process can be seen in the adjacent amino acid highlighter plot. The linear model relating the root-to-tip distances of pre-ART plasma HIV RNA sequences in this tree to their collection dates is shown in Fig. 3C, where the slope of this line represents the within-host pre-ART *env-gp120* evolutionary rate and the x-intercept represents the phylogenetically estimated infection date. Conditioning these metrics over all QC-passed trees revealed a mean root date of December 1995 (95% HPD, from August 1995 to March 1996), consistent with the participant’s clinically estimated infection date, and a mean *env-gp120* evolutionary rate of 4.8 × 10^−5^ (95% HPD 3.8 × 10^−5^ – 6.0 × 10^−5^) substitutions per nucleotide site per day (Table S1). All subsequent analyses were restricted to distinct HIV sequences per time point, as our goal was to examine the diversity and ages of distinct proviral sequences in a way that is not influenced by clonal expansion.

Beginning in October 2010, participant 1 experienced a prolonged viremia event that occurred after a documented change in ART regimen. Unfortunately, neither adherence nor drug resistance data were available to confirm the cause of the rebound. Nevertheless, this event allowed us to investigate the within-host evolutionary origins of HIV sequences rebounding in plasma, which we sampled at three time points between October 2010 and October 2011. All sequences sampled at the initial time point interspersed with plasma sequences that circulated in the 3 years prior to ART (Fig. 3B), where our phylogenetic methods dated these sequences to between late 2005 and the time of sampling (Fig. 3C and D). Rebound sequences subsequently sampled in April and October 2011 also dated to this era, which is expected given that many of these sequences were descendants of the initial rebounding population (Fig. S2 shows an enlarged portion of the phylogeny, where likely descendants are indicated by green arrows). Nevertheless, a monophyletic clade of closely related rebound sequences isolated in April 2011 was less divergent from the root and distinct from the rebound sequences sampled the previous October (Fig. S2, black bracket). This observation was consistent across all trees (see consistently earlier integration dates for these sequences in Fig. 3D). This suggests that these were not descendants of the initial rebounding population but rather descendants of a provirus or clonal infected cell population that reactivated independently near this time.

In striking contrast to the rebound HIV sequences, participant 1’s distinct proviral sequences subsequently sampled on ART in 2013, 2015, 2017, and 2018 interspersed throughout the entire phylogeny, nearly all the way back to the root (Fig. 3B). Though the inferred integration dates of these proviruses were slightly skewed toward the years leading up to ART, as well as the subsequent ART interruption period, numerous older proviruses were recovered, including one dating to September 1996, less than a year following infection (Fig. 3D). The integration date distributions of distinct proviruses sampled longitudinally on ART were highly stable over time (Kruskal-Wallis *P* = 0.6; Fig. 3D). Notably, these proviruses were overall significantly older than those that had previously rebounded in plasma (Mann-Whitney *P* = 0.0005 when comparing the ages of all proviruses vs. those of the initially rebounding population; *P* < 0.0001 when comparing all proviruses to the entire rebounding population). We also observed proviruses that were identical or near identical to sequences that had previously rebounded in plasma (Fig. S2, black arrows), consistent with reservoir re-seeding during this rebound event.

We next investigated whether the distinct proviral sequences sampled during ART showed any evidence of changing population genetic structure over time. This could happen, for example, if distinct within-host lineages were being eliminated during the initial years of ART or if proviral populations sampled at different time points were distinct from one another (which could occur, for example, if reservoir cells re-entered blood from a compartmentalized tissue population). Neither test revealed significant evidence of population structure: AMOVA yielded *P* = 0.3, while the CC test, conditioned over all passing trees, yielded a mean *P* = 0.16 (Fig. 3E). Therefore, even though proviral clonality increased during the first 9 years of ART (Fig. 2C), the number and composition of distinct proviral lineages remained consistent over time. In contrast, plasma rebound HIV sequences were significantly compartmentalized compared with the overall proviral pool (*P* = 0 for both AMOVA and CC), indicating that only a restricted subset of persisting proviruses re-seeded viremia at that time.

Within-host recombinants, which are an important source of within-host HIV diversity (36, 37), cannot be dated using the phylogenetic approaches used here. For recombinants, we instead used RDP4 (38) to obtain the origin date of each parental sequence component. We were particularly interested in the possibility of recombination between sequences from different infection eras, a phenomenon that has been predicted by mathematical modeling (39). The existence of such recombinants would support the notion that reservoir cells can be superinfected with HIV from another within-host era.

**Fig 4:**
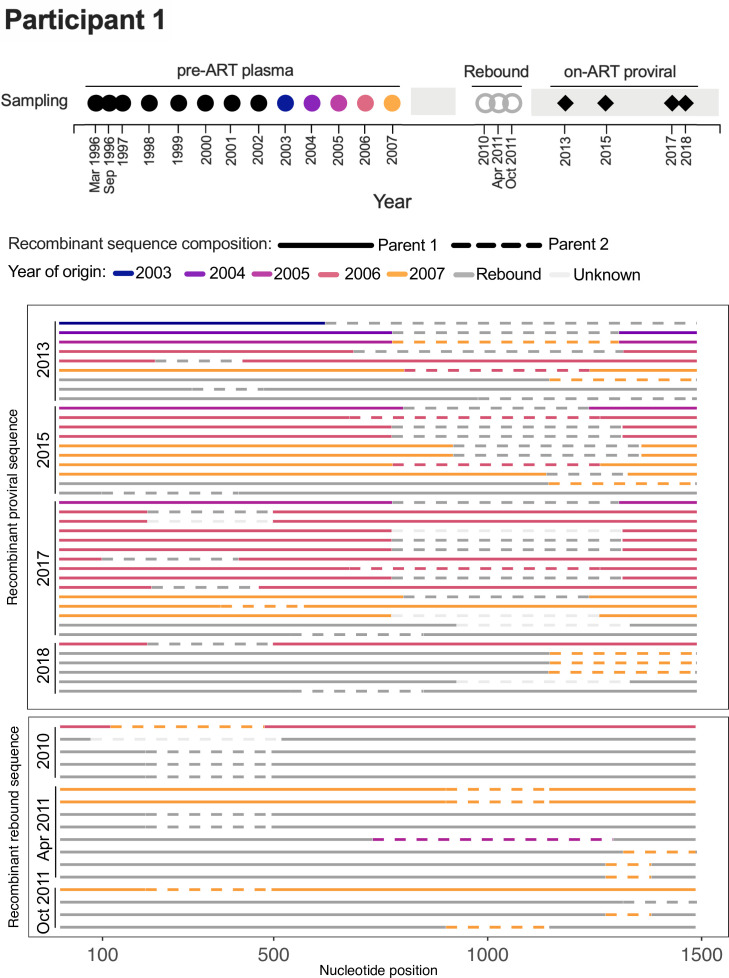
Participant 1: recombinant *env-gp120* proviral and rebound sequences. Colored circles in the sampling timeline (top) denote the year of origin of one or more recombinant sequence fragments detected among provirus or rebound HIV sequences (shown below). This timeline also shows the collection date of the sampled rebound HIV sequences (gray doughnuts) and longitudinal on-ART proviral sequences (black diamonds). The sampled recombinant sequences are grouped by type (proviral or rebound HIV) and year of collection, one sequence per line. The solid and dotted line fragments represent the two parent sequences, colored by year of origin.

In participant 1, we identified 40 distinct recombinant *env-gp120* proviral sequences that were sampled on ART between 2013 and 2018 (Fig. 4). None contained a parent sequence from the first 7 years of infection. Rather, 5 were mosaics of sequences that rebounded during the 2010–2011 treatment interruption, 6 were mosaics of sequences that circulated in the 3 years prior to ART, and 22 were mosaics of rebound sequences and pre-ART sequences from 2005 to 2007, which fueled the rebound event. The final five were sequences whose minor parent could not be identified. Two mosaics comprising 2003 and rebound sequences were observed, but these parent sequences could plausibly have circulated at the same time, as the root-to-tip divergences of sequences from these periods overlapped one another (see Fig. 3C). As such, there was no convincing evidence in this participant of recombinants between viruses from substantially different infection eras. Similarly, the 17 recombinants identified among the 2010–2011 plasma rebound sequences were all mosaics of rebound sequences and/or pre-ART sequences that fueled the rebound (Fig. 4).

### Participant 2

**Fig 5:**
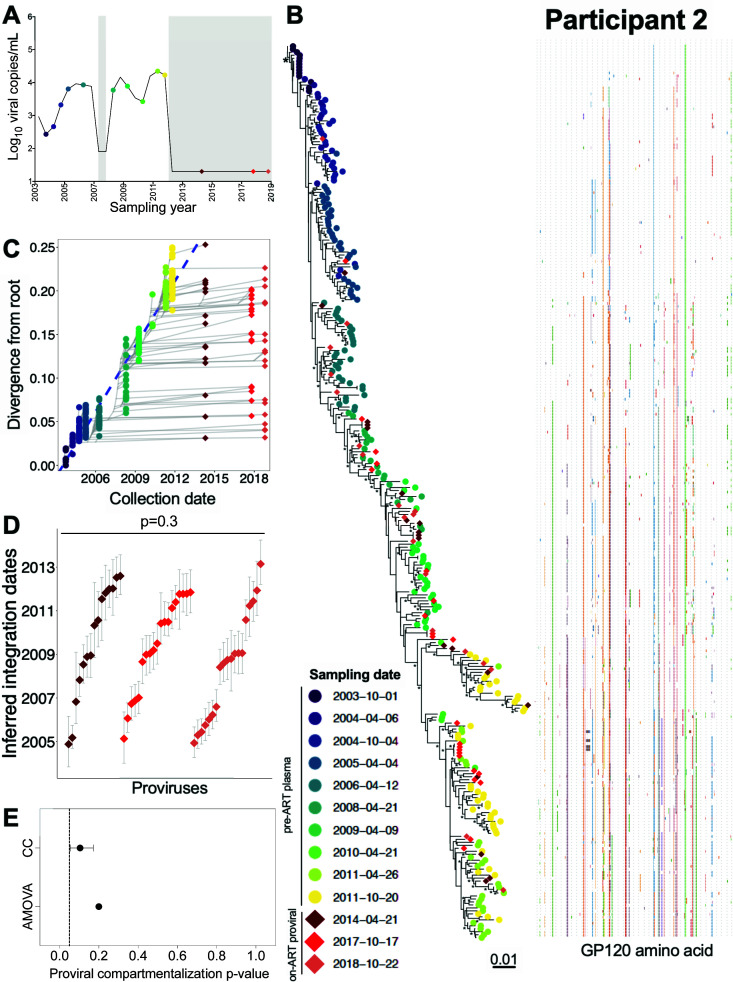
Participant 2: diversity and inferred integration dates of HIV sequences persisting during ART. Legend as in Fig. 3, except that **E** shows *P*-values from genetic compartmentalization tests applied to serially sampled proviruses only.

Participant 2 was estimated to have acquired HIV in January 2003 (Table 1). Though ART was briefly initiated in 2007, durable suppression was not achieved until ART was re-initiated in January 2012 (Table 1; Fig. 5A). We inferred 5,250 phylogenies from 239 plasma HIV RNA *env-gp120* sequences isolated during untreated infection, along with 61 proviral *env-gp120* sequences sampled at 2, 6, and 7 years post-ART. Of these, 3,842 trees passed QC, yielding a mean root date of May 2003 (95% HPD, from January to September 2003; Table S1). Proviral sequences sampled during ART interspersed throughout nearly the whole tree (example phylogeny and root-to-tip divergence plot in Fig. 5B and C), where the oldest provirus dated to late 2004, approximately 2 years after infection (Fig. 5D). The integration date distributions of distinct proviruses sampled over 7 years on ART remained consistent over time (Kruskal-Wallis *P* = 0.3; Fig. 5D) and showed no evidence of temporal population structure (AMOVA *P* = 0.2; CC mean *P* = 0.1; Fig. 5E).

Four recombinant proviral sequences were recovered from participant 2, three of which were mosaics of sequences that circulated in the 2 years prior to ART (Fig. S3A). In contrast, one recombinant had a 5′ half that dated to 2006 and a 3′ half that dated to 2010 (Fig. S3A; asterisk). Of note, the root-to-tip divergence measurements of the plasma HIV RNA sequences isolated in 2006 and 2010 did not overlap one another (see Fig. 5C), suggesting that sequences with such distinct divergence measurements could not have co-circulated during ongoing HIV evolution. Instead, the recovery of this recombinant sequence suggests that a reservoir cell dating to 2006 became superinfected with a sequence circulating in 2010 (or that a cell infected in 2010 became superinfected with HIV reactivated from 2006). This superinfected cell would then have needed to produce an infectious virion carrying both parent genomes, which then yielded a novel recombinant provirus upon infection of a new cell.

### Participant 3

**Fig 6:**
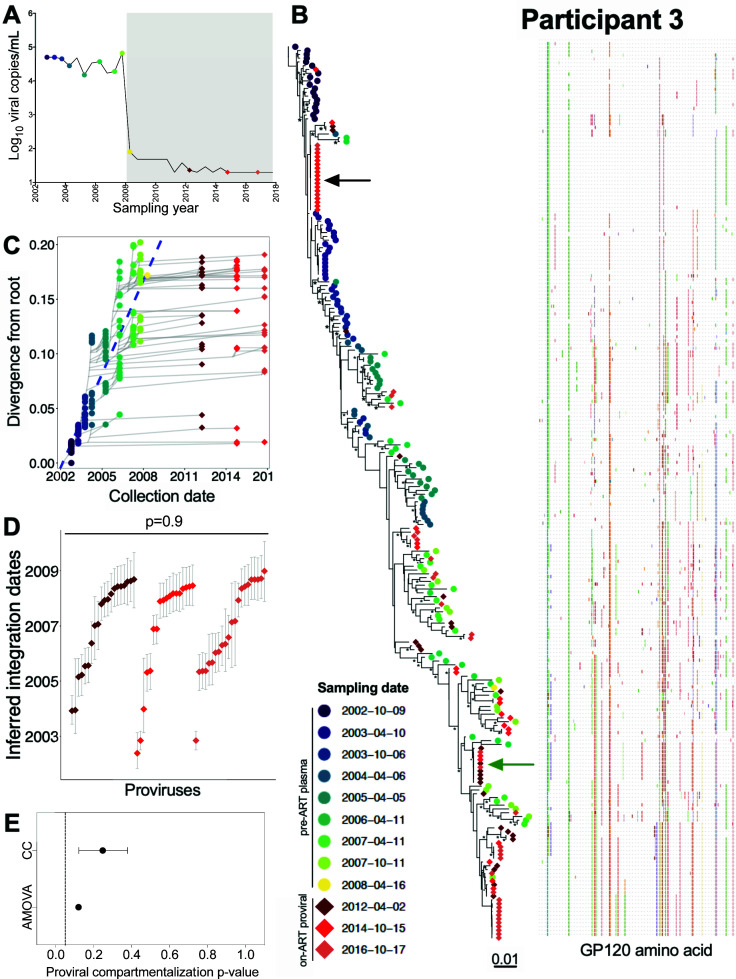
Participant 3: diversity and inferred integration dates of HIV sequences persisting during ART. Legend as in Fig. 5.

Participant 3 was estimated to have acquired HIV in July 2002 and initiated ART in January 2008 (Table 1; Fig. 6A). We inferred 4,500 phylogenies from 140 pre-ART plasma *env-gp120* sequences, published in (32), along with 96 proviral sequences sampled after 4, 7, and 9 years on ART. All passed QC, yielding a mean root date of November 2001 (95% HPD, July 2001 to March 2002), which was slightly earlier than the clinically estimated infection date of July 2002 (Table S1). Again, distinct proviral sequences sampled on ART interspersed throughout the tree (example phylogeny and divergence plot shown in Fig. 6B and C). We also observed a number of expanded clones. These included one relatively near the root that we recovered at both the second and third on-ART sampling time points (Fig. 6B, black arrow) and another more divergent one that we recovered at all on-ART time points (Fig. 6B, green arrow). The oldest recovered provirus, isolated in 2014, was estimated to have integrated in May 2002 (Fig. 6D). The integration date distributions of distinct proviruses were stable during ART (Kruskal-Wallis *P* = 0.9; Fig. 6D), with no evidence of changing population structure (AMOVA *P* = 0.1; CC mean *P* = 0.25; Fig. 6E). All 12 recombinant proviral sequences collected during ART were mosaics of sequences that circulated in the 3 years prior to ART (Fig. S3B).

### Participant 4

**Fig 7:**
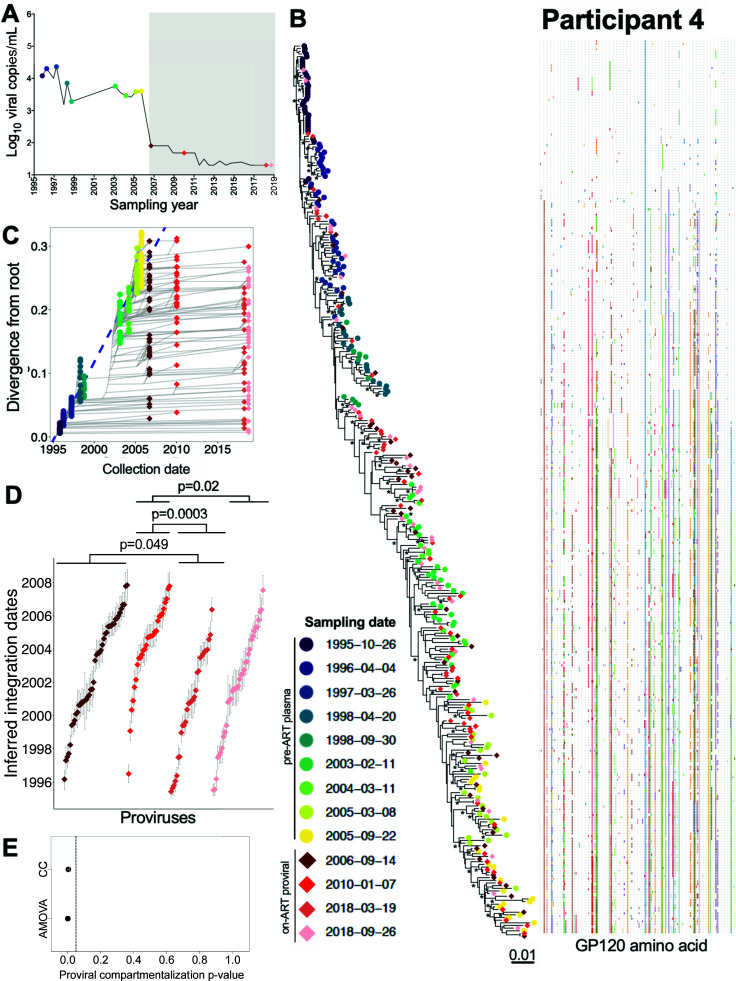
Participant 4: diversity and inferred integration dates of HIV sequences persisting during ART. Legend as in Fig. 5, except that **D** shows pairwise comparisons between groups after correction for multiple comparisons, as the overall Kruskal-Wallis test returned *P* = 0.0004.

Participant 4 was estimated to have acquired HIV in July 1995 and initiated suppressive ART in June 2006 (Table 1; Fig. 7A). We inferred 6,000 within-host phylogenies from 195 pre-ART plasma *env-gp120* sequences along with 141 proviral *env-gp120* sequences sampled at four time points up to 12 years post-ART (Table 1). All trees passed QC (example phylogeny and divergence plot in Fig. 7B and C), yielding a mean estimated root date of February 1995 (95% HPD, from October 1994 to June 1995; Table S1), which was only slightly earlier than the clinically estimated infection date. Proviruses isolated during ART had integrated throughout the entire course of untreated infection and included five distinct sequences, isolated in March and September 2018, that dated to 1995 (i.e., the first 6 months after transmission). In contrast to participants 1–3, the age distributions of distinct proviruses sampled in March and September 2018 (~12 years after ART initiation, the longest follow-up of any participant in the study) were on average older than those sampled in the earlier years of ART (Kruskal-Wallis *P* = 0.0004; pairwise post-test *P*-values shown in Fig. 7D). Both AMOVA (*P* = 0.001) and CC (mean *P* = 0.004) also supported a change in proviral population structure over this period (Fig. 7E).

Eleven recombinant proviral sequences were recovered from participant 4, 10 of which were mosaics of sequences that circulated in the 3 years prior to ART initiation (Fig. S4A). Of note, and similar to participant 2, one recombinant was a mosaic of a sequence from the year 2000 and another from 2005 (Fig. S4A; asterisk). The root-to-tip divergence measurements of plasma HIV RNA sequences circulating in 2000 and 2005 would not have been expected to overlap (see Fig. 7C), again supporting the notion that this recombinant arose as a result of a reservoir cell becoming superinfected with HIV from different infection eras.

### Participant 5

**Fig 8:**
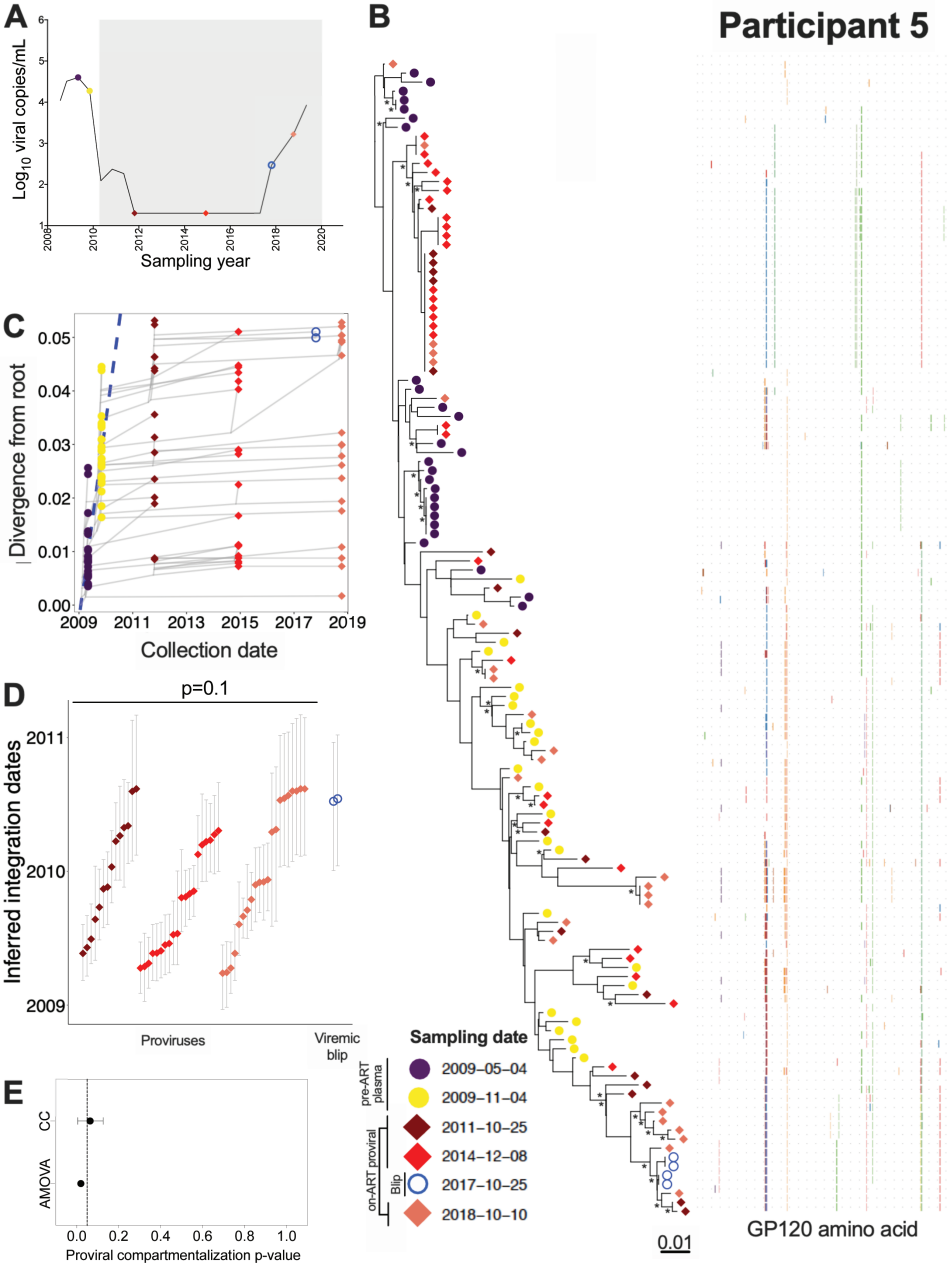
Participant 5: diversity and inferred integration dates of HIV sequences persisting during ART. Legend as in Fig. 5.

Participant 5 acquired HIV around March 2008 and initiated ART in February 2010 (Table 1; Fig. 8A). We inferred 1,500 phylogenies relating 50 *env-gp120* sequences collected at two pre-ART time points, 48 proviral *env-gp120* sequences collected at 2 and 5 years after ART initiation, 4 HIV RNA *env-gp120* sequences collected in year 8 when viremia initially rose to 295 HIV RNA copies/mL, and 26 proviral sequences collected in year 9, after viral control was lost (Table 1). Unfortunately, neither drug resistance nor adherence data were available to confirm the cause of the viremia control loss, and there were no documented ART regimen changes around this time. All phylogenies passed QC (Table S1) and yielded a mean estimated root date of December 2008 (95% HPD, October 2008 to February 2009), suggesting that we did not reconstruct all the way back to transmission (example phylogeny and divergence plot in Fig. 8B and C). Proviruses sampled during ART integrated throughout untreated infection, with the earliest dating to May 2009, about a year after transmission. In contrast, and similar to the observations from participant 1, both distinct plasma HIV RNA sequences isolated during the initial loss of viremia control dated to around ART initiation (Fig. 8B). Although the age difference between the emerging plasma viruses and the overall proviral pool did not reach statistical significance due to small numbers (*P* = 0.11), the emerging plasma viruses were clearly younger (Fig. 8D). The integration date distributions of proviruses sampled after 2, 5, and 9 years of ART were not significantly different from one another (Kruskal-Wallis *P* = 0.1), though the year 9 proviral pool featured a slightly larger number of younger proviruses than the first two time points, likely due to the loss of viremia control at this time (Fig. 8D). The *P*-values of the population structure tests suggested a similar shift (AMOVA yielded *P* = 0.02 and CC yielded a mean *P* = 0.06; Fig. 8E), though these observations did not reach our pre-defined significance requirement of *P* < 0.05 on both tests. Overall, these results indicated that participant 5’s proviral pool was largely stable in terms of genetic composition, though the recently emerging, younger HIV sequences in plasma may have re-seeded the reservoir to a modest extent. No recombinant sequences were recovered from this participant.

### Participant 6

**Fig 9:**
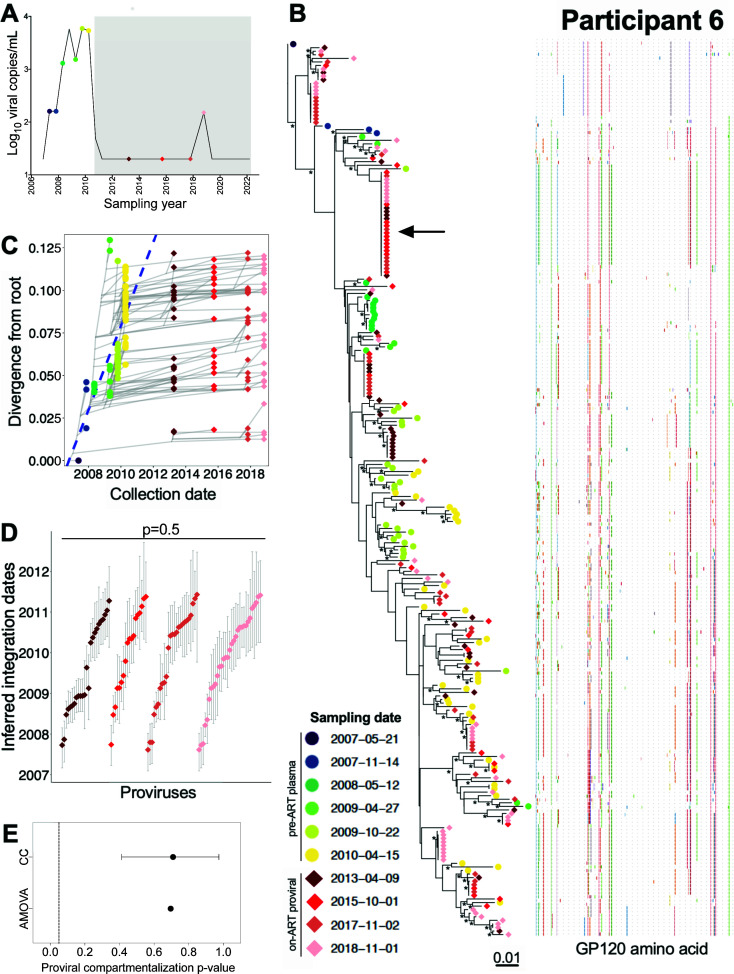
Participant 6: diversity and inferred integration dates of HIV sequences persisting during ART. Legend as in Fig. 5.

Participant 6 acquired HIV around August 2006 and initiated ART in July 2010 (Table 1; Fig. 9A). We inferred 3,000 phylogenies relating 80 plasma *env-gp120* sequences isolated at six time points during untreated infection, and 161 proviral *env-gp120* sequences collected at 3, 5, 7, and 8 years post-ART. Of these, 2,528 passed QC and yielded a mean root date of January 2007 (95% HPD, from September 2006 to May 2007), which was only slightly later than the clinically estimated root date (Table S1). Proviruses persisting on ART interspersed throughout the whole tree, with the oldest dating to March 2007 (Fig. 9B). We observed a number of long-lived clones, including one that was recovered at every on-ART time point (Fig. 9B, black arrow). Proviral age distributions remained consistent over time on ART (Kruskal-Wallis *P* = 0.5; Fig. 9D), with no evidence of changing population structure (AMOVA *P* = 0.7; CC mean *P* = 0.7; Fig. 9E). Only one recombinant proviral sequence was isolated from participant 6, whose parents dated to the 2 years prior to ART (Fig. S4B).

### On-ART proviral integration dates: cross-participant summary

**Fig 10:**
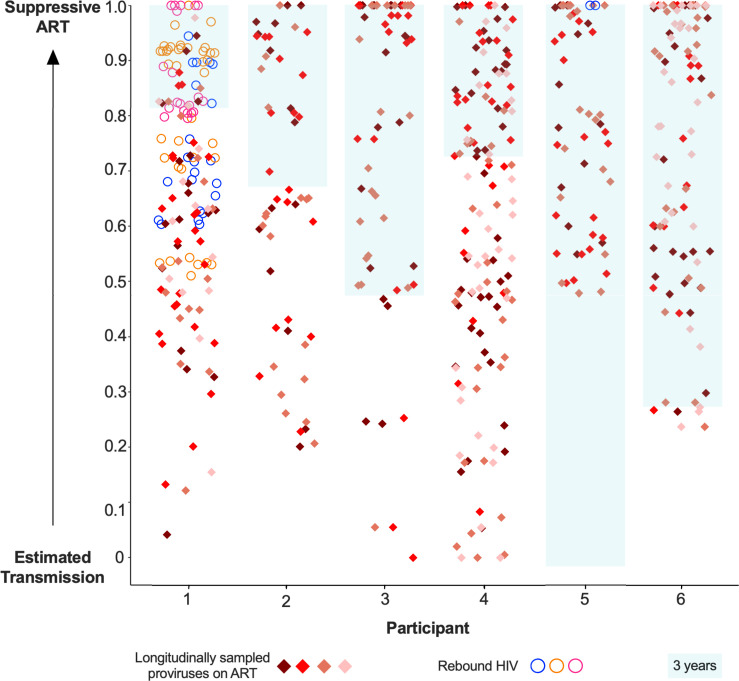
Scaled integration timings of on-ART proviral sequences and rebound HIV. Estimated integration timings of proviruses sampled longitudinally on-ART (diamonds) and rebound viruses (circles), depicted on a scale between the estimated date of infection and ART *suppression*. For participant 1, the date of ART re-suppression post-rebound was used. Sequences with integration dates after ART suppression are plotted at 1.0. For context, the blue-shaded box marks the 3 years leading up to ART suppression.

Plotting the inferred integration dates of all rebound HIV and on-ART proviral sequences on a scaled timeline ranging from estimated transmission to ART-mediated suppression allowed all participants to be visualized together (Fig. 10). These data clearly show that proviruses sampled on ART span a wide age range, which in some cases comprises the entirety of untreated infection (e.g.*,* participants 1, 3, and 4). The figure also shows that rebound viruses, shown as circles for participants 1 and 5, represent a younger subset of the overall persisting proviral pool (Mann-Whitney *P* = 0.0001).

### Sensitivity analysis 1: phylogenetic inference using *gag*

Though *env-gp120* is commonly used for within-host evolutionary studies (6, 9, 10, 40), evolution in this region is characterized by (sometimes dramatic) genetic bottlenecks largely imposed by the evolving antibody response (41). To confirm that our choice of HIV region did not unduly influence our results, we additionally dated participant 1, 3, and 7’s data sets using *gag*, as pre-ART *gag* sequences had previously been collected for these individuals (32).

We begin with participant 7, who was not included in the primary analysis because proviruses were only sampled at a single on-ART time point, but whose sequences are nevertheless critical to this validation (Table 1; Fig. S5). We inferred phylogenies relating newly collected *env-gp120* and *gag* on-ART proviral sequences (amplified independently) to the existing pre-ART plasma HIV RNA sequences (32) (see Fig. S5B and C, for representative trees and divergence plots, and Table S1 for summary statistics). The age distributions of distinct proviruses sampled on ART did not significantly differ based on which gene was used for dating (Mann-Whitney *P* = 0.07; Fig. S5D). *Env-gp120* and *gag* proviral clonal profiles were also highly comparable, even though these regions were amplified separately (Fig. S5E). Of the five *gp120* recombinant proviral sequences recovered during ART in participant 7, none contained parents that dated to substantively different eras of infection (Fig. S6). No recombinant *gag* sequences were identified.

*Gag* analysis also corroborated the original observations for participants 1 and 3. For participant 1, the percentage clonality based on *gag* was 23% (versus 33% for *env-gp120*) while for participant 3, it was 39% (versus 58% for *env-gp120*). *Gag* clones also increased over time and were observed across multiple time points (Fig. S7). For participant 1, *gag* analysis also confirmed that plasma rebound HIV sequences were significantly younger than, and represented a distinctive population compared to, the proviral pool that persisted during ART (Fig. S8E; both AMOVA and CC mean *P* = 0). *Gag* analysis also corroborated the independent reactivation of a distinct and slightly more ancestral proviral lineage at the second rebound sampling time point in April 2011 (Fig. S8B; bracket). It also confirmed that persisting proviruses had integrated throughout the entire course of untreated infection and that proviral age distributions were stable over time on ART (Kruskal-Wallis *P* = 0.6) with no evidence of temporal population structure (AMOVA *P* = 0.6; CC mean *P* = 0.62) (Fig. S8D and E). For participant 3, *gag* analysis similarly confirmed that proviral ages were stable during ART (Kruskal-Wallis *P* = 0.8) with no evidence of temporal population structure (AMOVA *P* = 0.74; CC mean *P* = 0.97) (Fig. S9E).

Importantly, the integration date distributions of on-ART sequences that were inferred from *env-gp120* versus *gag* were highly comparable. The sole exception was participant 1’s initial rebounding population, where *env-gp120* analysis returned on average older integration dates than *gag* analysis (*P* = 0.005, Fig. S10A). Other than this, *env-gp120* and *gag* analyses produced similar proviral integration dates for all seven proviral time points analyzed for participants 1 and 3 and the remaining two rebound time points analyzed for participant 1 (all *P* ≥ 0.1, Fig. S10A to C). No recombinant *gag* sequences were identified in participants 1 and 3. Overall, these observations indicate that our findings are not majorly influenced by the HIV region studied.

### Sensitivity analysis 2: evolution in HIV coreceptor usage as a non-phylogenetic validation

As there is uncertainty in phylogenetic reconstruction, we also corroborated our findings using a “tree-free” approach. To do this, we leveraged the shifts in HIV coreceptor usage that occurred over time in participants 1, 2, and 4 (coreceptor shifts did not occur in the other participants). We inferred coreceptor usage from the V3 region of *env-gp120* sequences using the geno2pheno (coreceptor) algorithm, which assigns each sequence a false positive rate (FPR) between 0 and 100. The FPR represents the likelihood that a CCR5-using virus is misclassified as CXCR4-using (i.e., sequences with low FPR are more likely to be CXCR4-using). For participants 1 and 2, coreceptor usage shifted from CXCR4-using to CCR5-using during untreated infection (participant 1’s shift was previously documented (32)), while participant 4 had a minority CXCR4-using population in early infection that steadily became more dominant (Fig. S11).

The coreceptor usage results corroborated the phylogenetic ones. Consistent with the tree-based analysis, participant 1’s FPR distributions differed significantly between rebound HIV and proviruses persisting longitudinally on ART (Mann-Whitney *P* = 0.002 for comparison of all rebound viruses to all proviruses; *P* = 0.003 for comparison of initial rebound viruses to all proviruses). In contrast, yet also consistent with the tree-based analyses, proviral FPR distributions were stable over time in both participants 1 and 2 (Kruskal-Wallis *P* = 0.3 (Fig. S11A) and *P* = 0.2 (Fig. S11B), respectively)) but shifted over time in participant 4 (Kruskal-Wallis *P* = 0.02, where the average FPR was significantly higher at the latest time point compared with one of the earlier ones; post-test *P* = 0.04 Fig. S11C).

### Temporal stability of on-ART proviral diversity: cross-participant analysis

**Fig 11:**
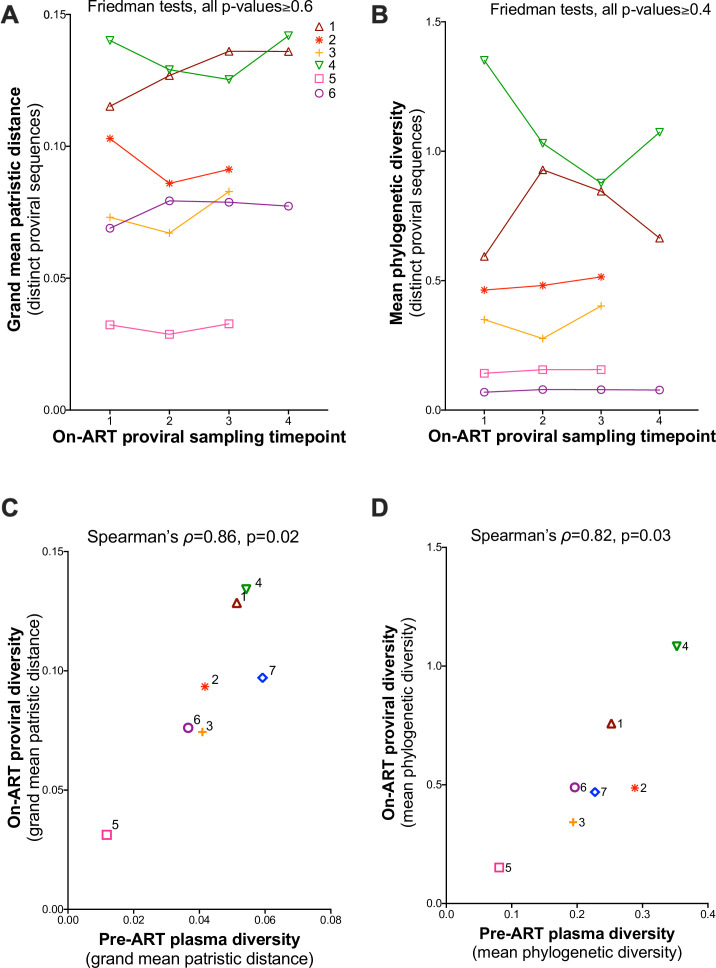
Proviral diversity during ART. (**A**) Grand mean within-host patristic distance separating all pairs of distinct proviral sequences per time point, with a line linking each participant’s values. (**B**) Same as A but expressed in terms of participants’ mean phylogenetic diversity of distinct sequences. *P*-values were computed using a Friedman test applied to the first three time points for all participants, as well as to all four time points for participants 1, 4, and 6. (**C**) Relationship between pre-ART plasma HIV RNA and on-ART proviral diversity, expressed in terms of grand mean patristic distance. (**D**) same as **C** but where diversity is expressed in terms of mean phylogenetic diversity.

As one of our objectives was to investigate proviral genetic stability (in terms of distinct lineages) during the initial years of ART, we concluded by testing for trends in overall within-host proviral diversity over time. We used two metrics: the grand mean patristic distance (Fig. 11A) and the mean phylogenetic diversity (Fig. 11B). Both metrics were computed from all distinct, non-recombinant proviral sequences isolated at each time point. For grand mean patristic distance, a Friedman test comparing the first three time points across all six participants yielded *P* = 0.6, while comparison across all four time points for participants 1, 4, and 6 yielded *P* = 0.7 (Fig. 11A). The corresponding values for mean phylogenetic diversity were *P* = 0.4 and *P* = 0.7 (Fig. 11B). These observations further support the notion that proviral genetic diversity is stable during the initial years of ART.

Of note, the diversity of distinct proviral lineages persisting on ART correlated strongly with the overall plasma HIV diversity generated during untreated infection: when measured as grand mean patristic distance, Spearman’s correlation yielded ρ = 0.86, *P* = 0.02 (Fig. 11C), and when measured as mean phylogenetic diversity, Spearman’s correlation yielded ρ = 0.82, *P* = 0.03 (Fig. 11D).

## DISCUSSION

We reconstructed within-host HIV evolutionary histories from pre-ART plasma and on-ART proviral sequences sampled over a median of 14 (range 9–23) years in seven participants. These analyses can reveal the lineage origins—and ages—of proviruses persisting on ART, as well as insights into the temporal stability of the on-ART proviral pool in terms of its genetic diversity, composition, and age distribution. Consistent with previous reports, clonal (defined as *env-gp-120*-identical) sequences persisted long-term—in fact, one clone was recovered at all time points over an 8-year period in participant 5. Clones also “waxed and waned” over time (e.g., participants 5 and 6) and dominated in some cases (participants 3 and 6’s proviral pools were >50% clonal) (20, 21, 42–48). To avoid our genetic diversity assessments being influenced by clonal expansion, which increased over time in 4 of 6 participants, clones were collapsed down to a single representative per time point.

Despite increasing clonality, the distinct proviral sequences comprising the proviral pool were highly stable in terms of genetic diversity, composition, and age distribution. Though we found no broad evidence that proviral diversity was being lost over time, we did detect a modest yet statistically significant shift in proviral composition in participant 4, for whom the proviruses sampled 12 years post-ART were on average older and exhibited different population structure than those sampled in the earlier years of ART. We do not believe that this is a sampling artifact, as proviruses were sampled twice in year 12 of ART, with consistent results. Rather, the more plausible explanation is that younger proviruses (i.e., those seeded just prior to ART initiation) were preferentially eliminated during the initial years of ART. This gradually shifted the balance towards older, more long-lived proviruses, making them more likely to be detected using the limiting-dilution approaches used here. This observation is also consistent with a study of four individuals with HIV subtype C with longitudinal on-ART sampling (6), as well as a recent study in a non-human primate model of HIV (49), both of which suggested that younger proviruses were preferentially eliminated during these initial years of ART. Of note, participant 4 had the longest follow-up of any individual in the study, which may have allowed the opportunity to observe this phenomenon.

Our study also indicates that the replication-competent HIV reservoir in blood (measured as HIV sequences that emerged in plasma post-ART in participants 1 and 5) represents a genetically restricted subset of the overall proviral pool, which is predominantly defective. Consistent with prior studies (5–7, 9–12), participants’ on-ART proviral pools ranged from modestly (e.g., participant 4) to substantially (e.g., participant 3) skewed towards viral variants that dated to the years immediately preceding ART, which is consistent with continual reservoir seeding—yet relatively rapid turnover—during untreated infection. By contrast, the plasma HIV sequences that emerged post-ART were a restricted subset that exclusively dated to the years immediately prior to ART. This suggests that replication-competent reservoir sequences older than this had already been eliminated, or were extremely rare. Alternatively, it is possible that they exist but could not reactivate (e.g., due to integration into inaccessible chromatin (50)) or that they reactivated but could not replicate effectively (e.g., because they were inhibited by host immune responses). Indeed, it is increasingly being appreciated that HIV rebound is a selective process, where the viruses that replicate to high levels in plasma are not necessarily those that reactivated first, but those that host immune responses, particularly antibodies, fail to control (51–53). By definition, the observation that rebound viruses integrated near the time of ART initiation means that they will be enriched in immune escape mutations, because sequences from this infection era will have had the longest time to adapt to within-host responses (30).

Participant 1’s data also suggested that, during extended ART interruption, viral rebound occurs in sequential “waves” of reactivation from individual reservoir cells (or clonal populations). This was supported by the emergence of slightly more ancestral viral sequences 6 months into the treatment interruption. Participant 1’s data are also consistent with a Simian Immunodeficiency Virus (SIV) study that showed that rebound viruses can re-seed the reservoir if rebound viral loads reach pre-ART levels (54), which occurred in this case.

Of note, recombinant proviruses were identified in nearly all participants, and recombinant HIV RNA sequences also emerged in plasma in participant 1 after ART interruption. Almost all of these recombinant sequences represented mosaics of sequences that plausibly co-circulated at the same time. Nevertheless, we identified two recombinant proviruses, one each from participants 2 and 4, whose parents dated to different enough infection eras that co-circulation of these sequences was unlikely. Rather, the discovery of these two proviruses suggests that reservoir cells can become superinfected with HIV from another infection era. Though mathematical modeling suggests that this type of recombination occurs, and represents a latent HIV genome survival mechanism (39), it has never to our knowledge been empirically observed. We acknowledge however that our observations are not definitive and that HIV sequences with substantially different root-to-tip divergences could theoretically have co-circulated for long periods yet remained unsampled in blood.

The source of recombinant viruses during rebound also remains an open question (31). While recombinant plasma HIV RNA sequences were observed during participant 1’s rebound event, we did not identify any proviruses that exactly matched these sequences (though proviral sampling occurred some years after the rebound). While this suggests that recombinants were generated *de novo* during rebound, we cannot exclude the possibility that matching proviruses did exist in blood but we failed to detect them, or that they had existed but were eliminated before we were able to sample them or that recombinant proviruses resided in tissue.

Our study has some caveats and limitations. All participants were women. Though there is no evidence that men and women differ in terms of rates of viral evolution (32) nor on-ART proviral genetic composition and age distribution (6), there is evidence that *ex vivo* reactivation potential and residual immune activation differ by sex (55–57). Due to very limited sample availability (only 10 million peripheral blood mononuclear cells (PBMCs) per proviral time point), we performed sub-genomic amplification. This is because near-full-genome HIV amplification would likely have generated many sequences with various large deletions in *env-gp120* (and/or *gag*) that could not be phylogenetically dated. We cannot therefore discriminate intact from defective proviruses. In fact, using data from another study (30), we estimate a 22% overall average likelihood (range 2%–35% depending on the participant) that an intact *env-gp120* sequence comes from a genomically intact provirus. Because we only sequenced part of the HIV genome, we also cannot definitively characterize proviruses as clonal, which would require full-genome sequencing and integration site characterization. We also acknowledge that sequences isolated only once may still be part of a clonal set (58). Because biological material was so limited, we isolated proviruses directly from PBMCs, so we could not quantify reservoir sizes nor identify the cell types harboring them. Despite these limitations, our study provides insights into the within-host evolutionary origins and temporal stability of proviral lineages on ART, along with the origins of HIV RNA emerging in blood. It also boosts the representation of women living with HIV subtype B, who are under-represented in the within-host HIV evolutionary reservoir dynamics literature.

In conclusion, the diversity of proviruses persisting on ART, which are largely genetically defective (13–15), broadly reflects the extent of within-host HIV evolution prior to ART (6, 7). Our results also reveal that the clonal expansion that commonly occurs during the initial years of ART is not appreciably accompanied by the loss of distinct proviral lineages during this time. In fact, on-ART proviral genetic composition remained remarkably stable, with the exception of participant 4, in whom some of the proviruses that had integrated near ART initiation had been preferentially eliminated by the 12th year of ART. Our analysis of recombinant sequences also supports the notion that reservoir cells can become superinfected with HIV reactivated from older infection eras, yielding mosaics of older and younger sequences. Finally, our observations suggest that the replication-competent reservoir (studied here as rebound HIV sequences) comprises a genetically restricted, younger subset of all proviruses persisting in blood. If so, HIV cure strategies will need to eliminate a reservoir whose key characteristics may differ from those of the overall proviral pool.

## MATERIALS AND METHODS

### Study population

The WIHS was a prospective, multi-center, cohort study of US women living with or without HIV (59–61). WIHS participants were recruited at 10 sites over four time periods starting in 1994. While the WIHS study ended in 2019, most participants continue to be followed under similar protocols as part of the MACS/WIHS Combined Cohort Study (MWCCS) (62). Eligibility criteria and study protocols for the WIHS have been previously described (59–61). Briefly, data were collected using structured in-person interviews and standardized physical and laboratory assessments, with study visits occurring every 6 months. Eligible women had documentation of reactive anti-HIV serology (and if positive a confirmatory test) or, if they were HIV-seronegative, had risk factor(s) for HIV exposure. Baseline sociodemographic characteristics and HIV risk factors were similar between HIV-seropositive and HIV-seronegative women.

We studied seven WIHS participants with the following criteria: documented HIV seroconversion during follow-up, initiated ART during chronic infection, at least four longitudinal pre-ART plasma samples available, and on-ART PBMC samples available. These seven participants represented all WIHS participants who met these criteria. HIV infection dates were calculated as the midpoint between last negative and first seropositive study visits. At the time of the last proviral sampling, participants were a median of 53 (range 49–54) years of age and had been receiving ART for a median of 9 years (range 2.8–12.3 years). In total, we collected longitudinal HIV RNA sequences over a median of 9 (range 2–13) study visits per participant spanning a median of 9 (range 0.8–11.8) years of untreated infection. Plasma HIV RNA sequences for participants 1, 3, and 7 were published previously (32). In addition, we collected single-genome-amplified proviral sequences over a median of 3 (range 1–4) time points per participant spanning a median of 8.7 (range 2.8–12.3) years on ART (only 10 million PBMCs per on-ART time point were available for analysis). We also isolated plasma HIV RNA sequences from participant 1 at three time points after ART was interrupted and from participant 5 when viral control was initially being lost.

### HIV amplification, sequencing, and curation

Total nucleic acids were extracted from plasma using the NucliSENS EasyMag (BioMerieux, Marcy-l'Étoile, France). If the plasma viral load (pVL) was <2,000 copies/mL, extracts were DNase I-digested (New England Biolabs) to minimize the risk of amplifying proviral DNA. Genomic DNA was extracted from 10 million PBMCs per time point using the QIAamp DNA Mini Kit (Qiagen). Single-genome amplification of a subgenomic HIV region (*env-gp120* and *gag* where applicable) was performed as follows. For plasma HIV RNA, cDNA (generated using NxtScript reverse transcriptase; Roche) was generated using HIV-specific primers and endpoint diluted such that subsequent nested PCR reactions (generated using the Expand High Fidelity PCR system; Roche) yielded no more than 30% positive amplicons (12, 46). Proviral DNA extracts were similarly endpoint diluted and amplified by nested PCR. For *env-gp120*, first round primers were as follows: 5′-TTAGGCATCTCCTATGGCAGGAAGAAGCGG-3′ (forward; HIV reference strain HXB2 genomic nucleotide coordinates 5957-5986) and 5′-TAAGTCATTGGTCTTAAAGGTACC-3′ (reverse; HXB2 9038-9015); second round primers were 5'- GGCCGCGTCGACAAGAGCAGAAGACAGTGGCAATGA-3′ (forward; HXB2 6194-6228) and 5′-GGCCGCGGATCCGTGCTTCCTGCTGCTCCCAAGAAC-3′ (reverse; HXB2 7823-7787) (32). For *gag*, first round primers were 5′-AAATCTCTAGCAGTGGCGCCCGAACAG-3′ (forward; HXB2 629-649) and 5′-TAACCCTGCGGGATGTGGTATTCC-3′ (reverse; HXB2 2849-2826); second round primers were 5′-GCAGGACTCGGCTTGCTGAA-3′ (forward; HXB2 691-710) and 5′-TATCATCTGCTCCTGTATC-3′ (reverse; HXB2 2343-2325). Negative controls were included in every run. For extracts from plasma samples with low pVL, we confirmed that HIV RNA amplification did not occur in the absence of reverse transcription. Amplicons were sequenced on a 3730xl automated DNA sequencer using BigDye (v3.1) chemistry (Applied Biosystems). Chromatograms were analyzed using Sequencher (v5.0/v5.4.6) (GeneCodes). Sequences with nucleotide mixtures were excluded from analysis.

Hypermutated sequences were identified using Hypermut (63). Sequences exhibiting evidence of putative within-host recombination were identified using RDP4 v4.1 (38). This program identifies the putative major and minor parent sequences for each recombinant, along with their approximate breakpoints, allowing us to date each sequence fragment by assigning the date of origin of its parent. Hypermutant and recombinant sequences, along with those with minor defects (e.g., small deletions), were retained in the analysis of clonality but excluded from phylogenetic inference. Sequence alignments were performed in a codon-aware manner using MAFFT v7.471 (64). Alignments were inspected and manually edited in AliView v1.26 (65). Following automated model selection using ModelFinder (66), *between-host* phylogenies were inferred by maximum-likelihood methods using IQ-TREE 2, with the ultrafast bootstrap option (1,000 bootstraps) (67, 68).

### Within-host phylogenetic inference and proviral dating

Within-host phylogenies were inferred from *env-gp120* and *gag* sequence alignments comprising all plasma and proviral sequences collected per participant. To mitigate uncertainty in phylogenetic reconstruction, we inferred a median of 4,500 (range 1,500–15,000) phylogenies per participant using Bayesian approaches and conditioned results across all trees. To do this, we first reduced each within-host nucleotide sequence alignment to distinct, intact, non-recombinant sequences and determined the best-fitting nucleotide substitution model for each alignment using jModelTest v2.1.10 (Table S1) (69). Next, Markov chain Monte Carlo (MCMC) methods were used to build a distribution of phylogenies per participant without enforcing a molecular clock. Two parallel runs with MCMC chains of a median of 30 million generations, sampled every 10,000 generations, were performed in MrBayes, v3.2.5 (70) using the best-fitting substitution model and model-specific or default priors. Convergence was assessed by ensuring the standard deviation of split frequencies was <0.04, and the effective sampling size of all parameters was ≥200 and by visual inspection of parameter traces using Tracer v1.7.2 (71). In the single case where convergence was not achieved (participant 1, *env-gp120*), the run was terminated at 100 million generations. The first 25% of MCMC iterations was discarded as burn-in, yielding a minimum of 1,500 (maximum 15,000) *env-gp120* and *gag* phylogenies per participant (Table S1).

We then inferred the integration dates of on-ART sequences of interest using a phylogenetic approach (7). First, each tree was rooted at the location that maximized the correlation between the root-to-tip distances of the pre-ART plasma HIV RNA sequences and their sampling dates, which represents the most recent common ancestor of the data set. We then fit a linear model relating the root-to-tip distances of pre-ART plasma HIV sequences to their collection dates, where the slope of this line represents the average within-host gene-specific evolutionary rate, and the x-intercept represents the root date. Model fit was assessed by comparing the model’s Akaike Information Criterion (AIC) to that of a null model with a zero slope. In order to pass QC, a phylogeny required a ΔAIC ≥ 10 and an inferred root date that was prior to the first plasma sampling. A median of 3,842 (range 1,278–7,218) phylogenies per participant passed QC (Table S1). The linear models from these QC-passed phylogenies were used to convert the root-to-tip distances of on-ART sequences of interest to their integration dates. The custom R script for this method is available at https://github.com/cfe-lab/phylodating. The script was implemented via GNU parallel (72) to run more than one tree at a time. The integration dates were then averaged across all QC-passed phylogenies per participant to produce mean integration date estimates and 95% HPD estimates, computed using R package HDInterval (version 0.2.2).

As phylogenies were inferred using distinct within-host HIV sequences only, identical sequences were then grafted back onto the phylogenies using the add.tips function in the R package phangorn, v2.8.1, as appropriate for each analysis (73). The example phylogeny shown for each participant was the highest likelihood tree among those that passed QC. Phylogenies and highlighter plots were plotted using the R (v4.1.2) package ggtree version 3.21. Node support values were derived from Bayesian posterior probabilities generated from the consensus trees.

### Proviral population genetic structure and diversity analyses

Within-host proviral populations sampled during ART were tested for evidence of population genetic structure using AMOVA (74), which is a genetic distance-based test, and the CC test (75), which is a tree-based test. These were chosen because they can test for population structure across more than two time points. Tests were performed on distinct sequences per on-ART time point. AMOVA was implemented in the R package pegas, v1.1 (76) using the K80 substitution model (77), where statistical significance was assessed via 1,000 permutation tests. CC test statistics were averaged over all QC-passed phylogenies per participant, using a custom R script available at https://github.com/brj1/HIVCompartmentalization. A data set was classified as having evidence of a population structure if both tests returned *P* < 0.05 (where, for CC, this was defined as a mean *P* < 0.05 over all QC-passed trees).

Within-host proviral diversity was quantified using two metrics: the grand mean patristic (tip-to-tip phylogenetic) distance, calculated as the mean patristic distance between all pairs of distinct sequences per time point averaged over all QC-passed phylogenies, and the mean phylogenetic diversity, calculated by summing the edge lengths of all distinct sequences per time point, averaged over all QC-passed phylogenies (78). Both metrics were computed using custom R scripts available at https://github.com/brj1/HIVCompartmentalization. All tests of significance were two tailed (except compartmentalization tests which are one tailed), with *P* < 0.05 denoting statistical significance. All other statistical analyses were performed in Prism, v9.0 (GraphPad Software).

### HIV coreceptor usage

Coreceptor usage was predicted from the V3 region of HIV-1 *env-gp120* sequences using geno2pheno (coreceptor) (https://coreceptor.geno2pheno.org) (79). This support vector machine-based approach assigns each V3 sequence a FPR that represents the probability of falsely classifying a CCR5-using virus as CXCR4-using. We considered V3 sequences with FPR < 10% as CXCR4-using and those with FPR ≥ 10% as CCR5-using.

## Data Availability

The nucleotide sequences reported in this paper are available in GenBank (proviral DNA: accession numbers OR403739–OR404029, OR404030–OR404055, OR404056–OR404819, and OR404820–OR404981; HIV RNA: accession numbers OR402899–OR403056 and OR403057–OR403738).

## SUPPLEMENTAL MATERIAL

Supplemental figures jvi.01655-23-s0001.pdf Figures S1 to S11.

Table S1 jvi.01655-23-s0002.pdf Bayesian within-host phylogenetic inference details.

ASM does not own the copyrights to Supplemental Material that may be linked to, or accessed through, an article. The authors have granted ASM a non-exclusive, world-wide license to publish the Supplemental Material files. Please contact the corresponding author directly for reuse.
