# Supplementary material for: The replication-competent HIV reservoir is a genetically restricted, younger subset of the overall pool of HIV proviruses persisting during therapy, which is highly genetically stable over time
Source: J Virol. 2024 Jan 12;98(2):e01655-23. doi: 10.1128/jvi.01655-23 (PMC10878278; doi:10.1128/jvi.01655-23)
Supplement: Supplemental figures — Figures S1 to S11. [file jvi.01655-23-s0001.pdf]

Supplemental Figures for

The replication-competent HIV reservoir is a genetically restricted, younger subset of the overall pool of HIV proviruses persisting during therapy, which is highly genetically stable over time

Aniqa Shahid[1,2], Signe MacLennan[1], Bradley R. Jones[2,3], Hanwei Sudderuddin[2], Zhong Dang[2], Kyle Cobarrubias[2], Maggie C. Duncan[1,2], Natalie N. Kinloch[1,2], Michael J. Dapp[4], Nancie M Archin[5], Margaret A. Fischl[6], Igho Ofotokun[7], Adaora Adimora[8], Stephen Gange[9], Bradley Aouizerat[10], Mark H. Kuniholm[11], Seble Kassaye[12], James I. Mullins[4,13,14], Harris Goldstein[15], Jeffrey B. Joy[2,3,16], Kathryn Anastos[17], Zabrina L. Brumme[1,2*], and the MACS/WIHS combined cohort study (MWCSS)

[1]Faculty of Health Sciences, Simon Fraser University, Burnaby, BC, Canada
[2]British Columbia Centre for Excellence in HIV/AIDS, Vancouver, BC, Canada
[3]Bioinformatics Program, University of British Columbia, Vancouver, BC, Canada
[4]Department of Microbiology, University of Washington, School of Medicine, Seattle, WA, USA
[5]UNC HIV Cure Center, Institute of Global Health and Infectious Diseases, University of North Carolina at Chapel Hill, NC, USA
[6]Department of Medicine, University of Miami School of Medicine, Miami, FL, USA
[7]Division of Infectious Diseases, Department of Medicine, Emory University School of Medicine, Atlanta, GA, USA
[8]Department of Epidemiology, UNC Gillings School of Global Public Health, University of North Carolina at Chapel Hill, Chapel Hill, NC, USA
[9]Department of Epidemiology, Johns Hopkins Bloomberg School of Public Health, Baltimore, MD, USA
[10]College of Dentistry, New York University, New York, NY, USA
[11]Department of Epidemiology and Biostatistics, University at Albany, State University of New York, Rensselaer, New York, NY, USA
[12]Division of Infectious Diseases and Tropical Medicine, Georgetown University, Washington, DC, USA
[13]Department of Global Health, University of Washington, School of Medicine, Seattle, WA, USA
[14]Department of Medicine, University of Washington, School of Medicine, Seattle, WA, USA
[15]Departments of Microbiology and Immunology and Pediatrics, Albert Einstein College of Medicine, Bronx, New York, NY, USA
[16]Department of Medicine, University of British Columbia, Vancouver, BC, Canada
[17]Department of Medicine, Albert Einstein College of Medicine, New York, NY, USA
*Corresponding author

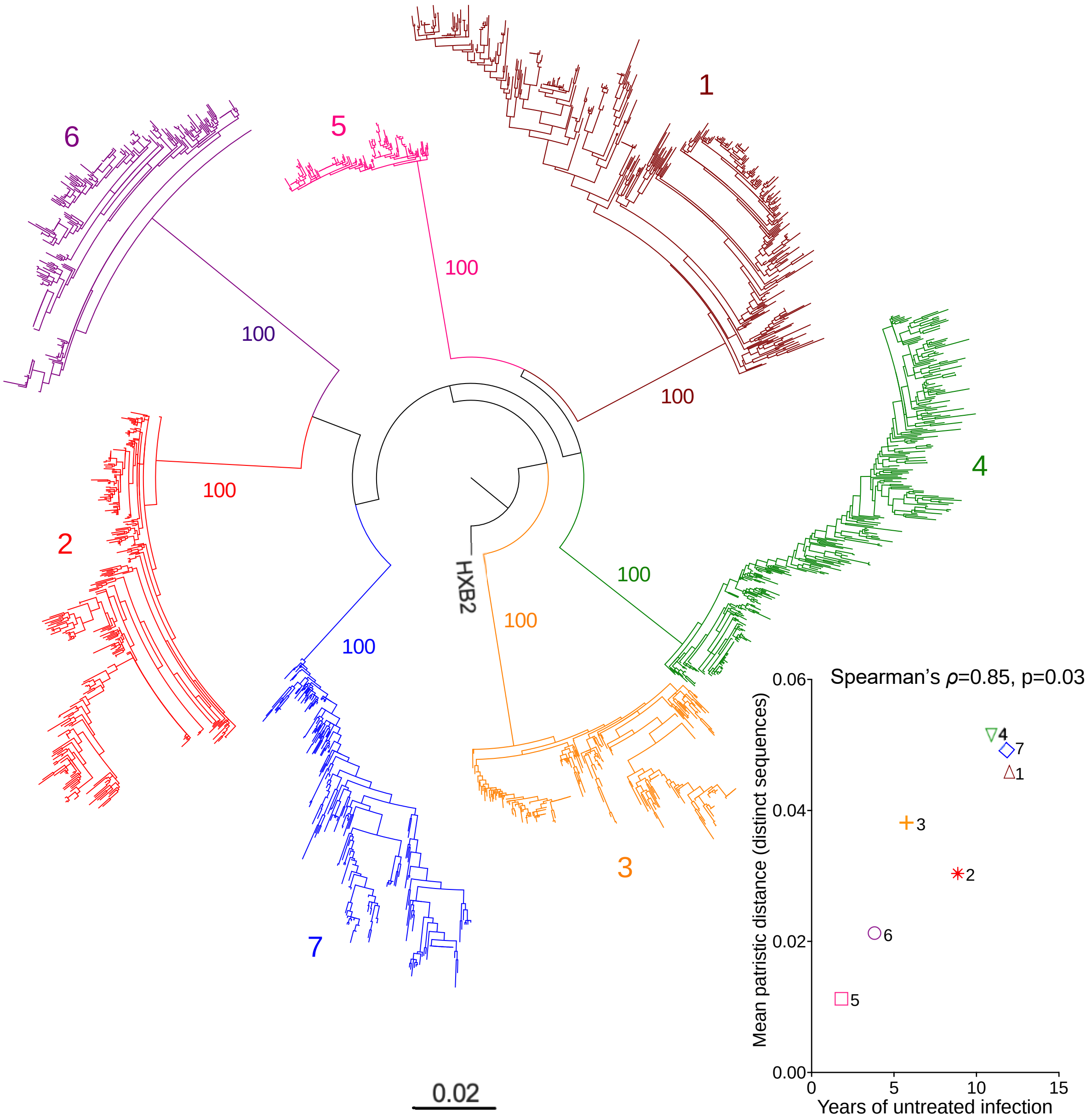

Fig. S1: Between-host HIV *env-gp120* phylogeny. Maximum-likelihood phylogeny inferred from all intact, non-hypermutated and non-recombinant *env-gp120* sequences, with each clade labeled by participant ID. Tree is rooted on the HIV subtype B reference strain HXB2. Numbers on internal branches indicate bootstrap values. Scale in estimated substitutions per nucleotide site. *Inset:* Correlation between overall within-host *env-gp120* diversity (calculated as mean patristic distance of distinct sequences) and years of untreated infection.

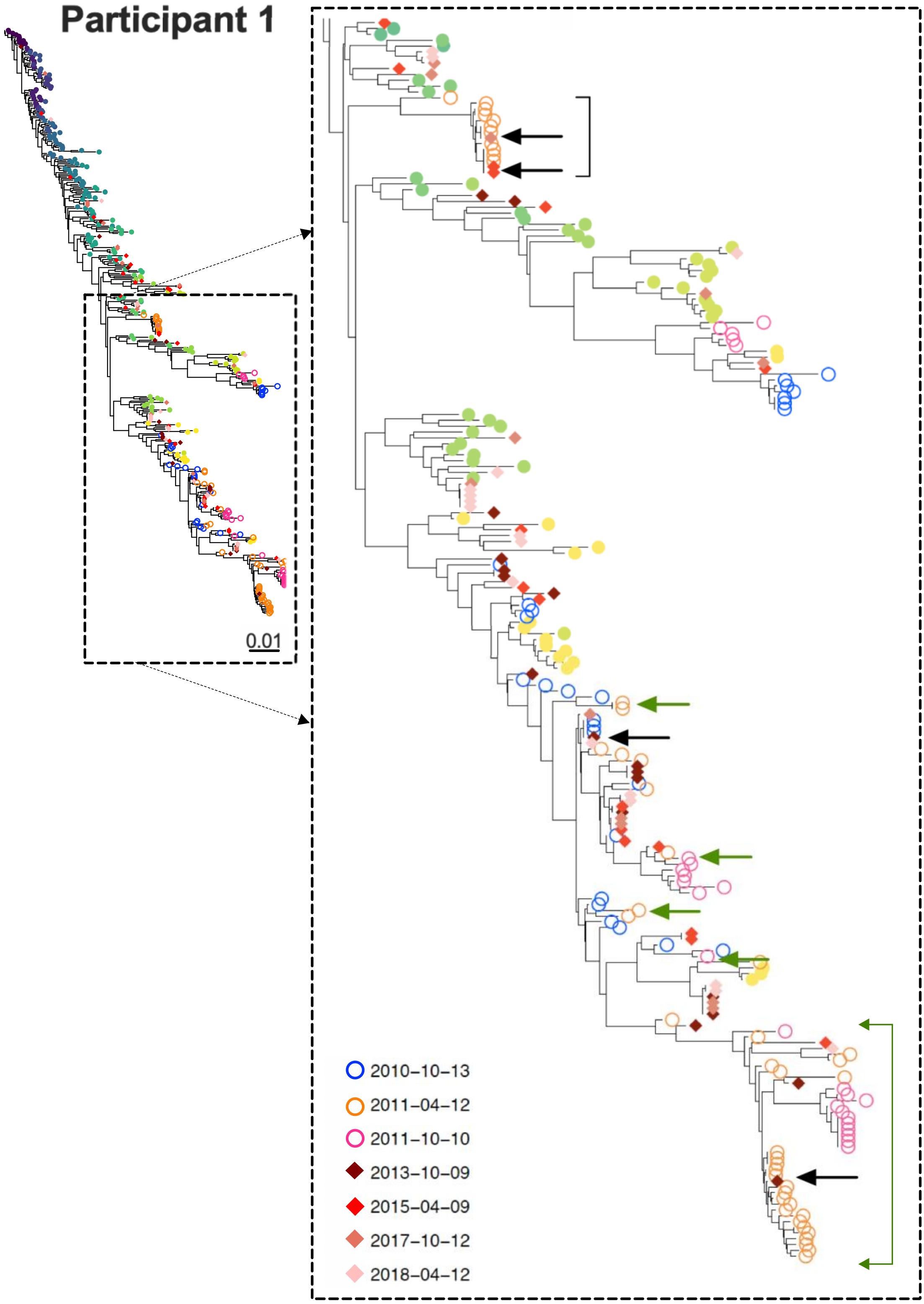

Fig. S2: Enlarged portion of participant 1's *env-gp120* within-host phylogeny. Green arrows and large green bracket point to examples of rebound viruses that could be descendants of variants that rebounded at prior time point(s). Small black bracket identifies the cluster of April 2011 rebound sequences that are more ancestral than those that initially rebounded, suggesting independent reactivation of this lineage. Black arrows point to proviruses identical or near identical to the rebound HIV, consistent with reservoir re-seeding during this rebound event.

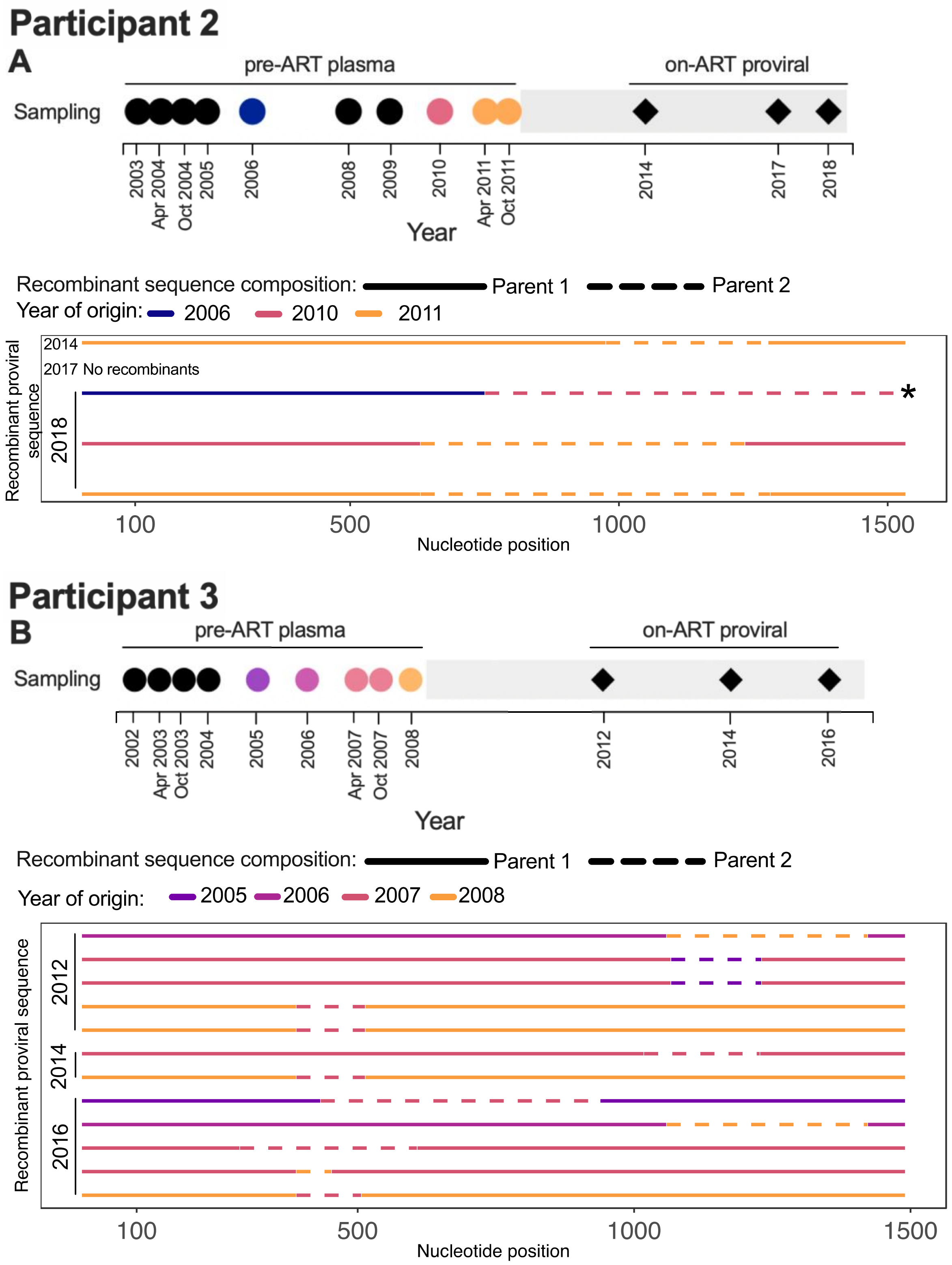

Fig. S3: Participants 2 and 3: recombinant *env-gp120* proviral sequences. (A) Colored circles in participant 2's sampling timeline (top) denote the year of origin of one or more recombinant proviruses (below). Recombinant proviruses are grouped by year of collection, with solid and dotted lines representing the two parent sequences, colored by year of origin. (B) same, but for participant 3.

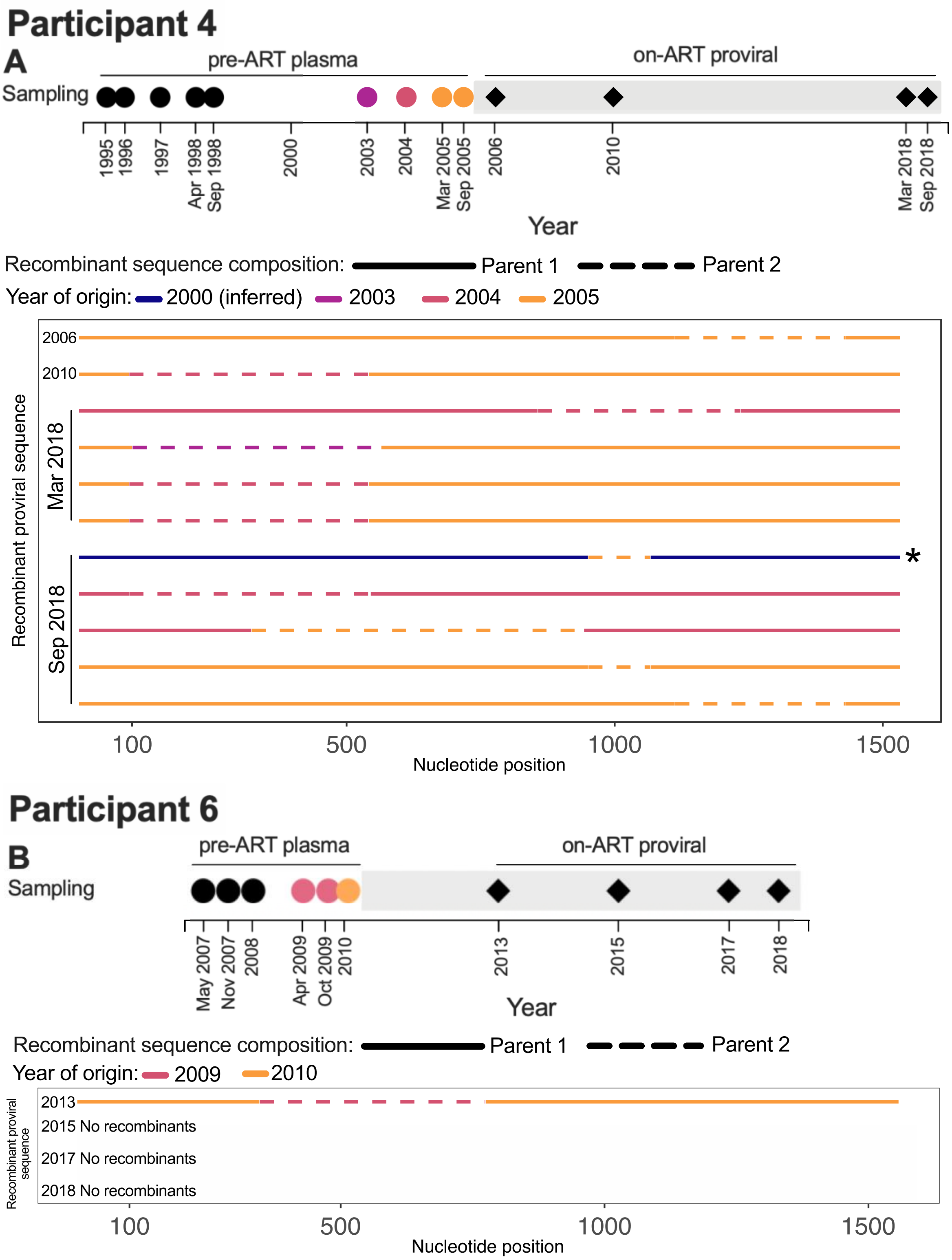

Fig. S4: Participants 4 and 6: recombinant *env-gp120* proviral sequences. Legend as in S3 Fig.

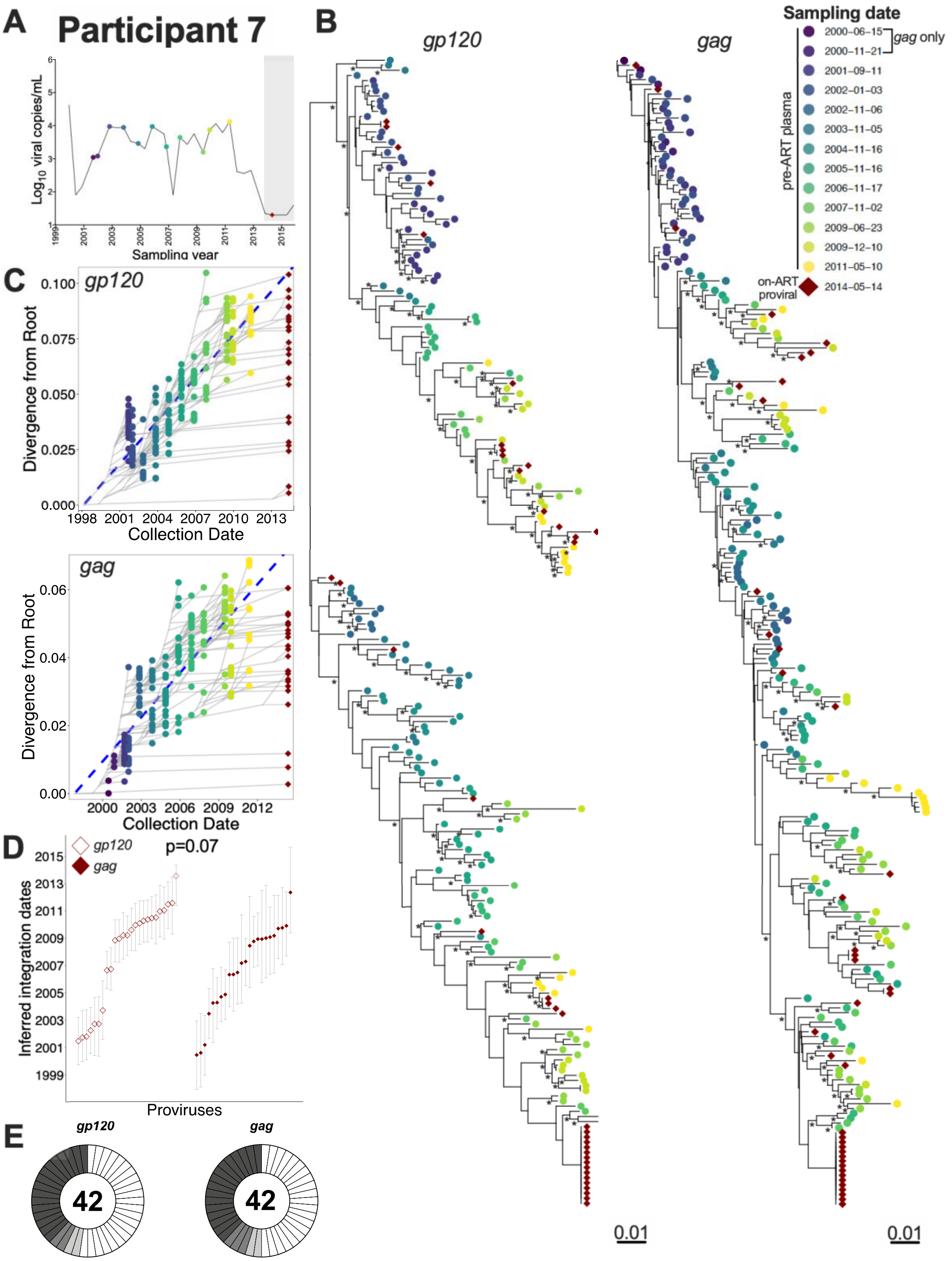

Fig. S5: Participant 7: diversity and inferred integration dates of HIV sequences persisting during ART. (A) Plasma viral load and sampling history. Circles denote pre-ART HIV RNA sampling, diamond denotes on-ART proviral sampling. Grey shading denotes ART. (B) Example rooted within-host *env-gp120* and *gag* phylogenies, with scale in estimated substitutions per nucleotide site. Asterisks identify nodes supported by posterior probabilities ≥70%. (C) Linear models

(dashed blue diagonal) derived from the trees shown in (B), that relate the plasma HIV RNA collection dates (colored circles) to their root-to-tip distance. This model is then used to convert the root-to-tip distances of distinct on-ART proviral sequences to their integration dates. Grey lines trace the phylogenetic relationships between sequences. (D) Inferred integration dates and associated 95% HPD intervals of *env-gp120* proviral sequences (open diamonds) and *gag* proviral sequences (closed diamonds) sampled on ART. P-value computed using the Mann-Whitney U-test. (E) Donut plots showing the total number of *env-gp120* and *gag* proviral sequences collected, where white slices denote sequences observed only once and those in various shades of grey identifying sequences observed more than once (i.e., clones).

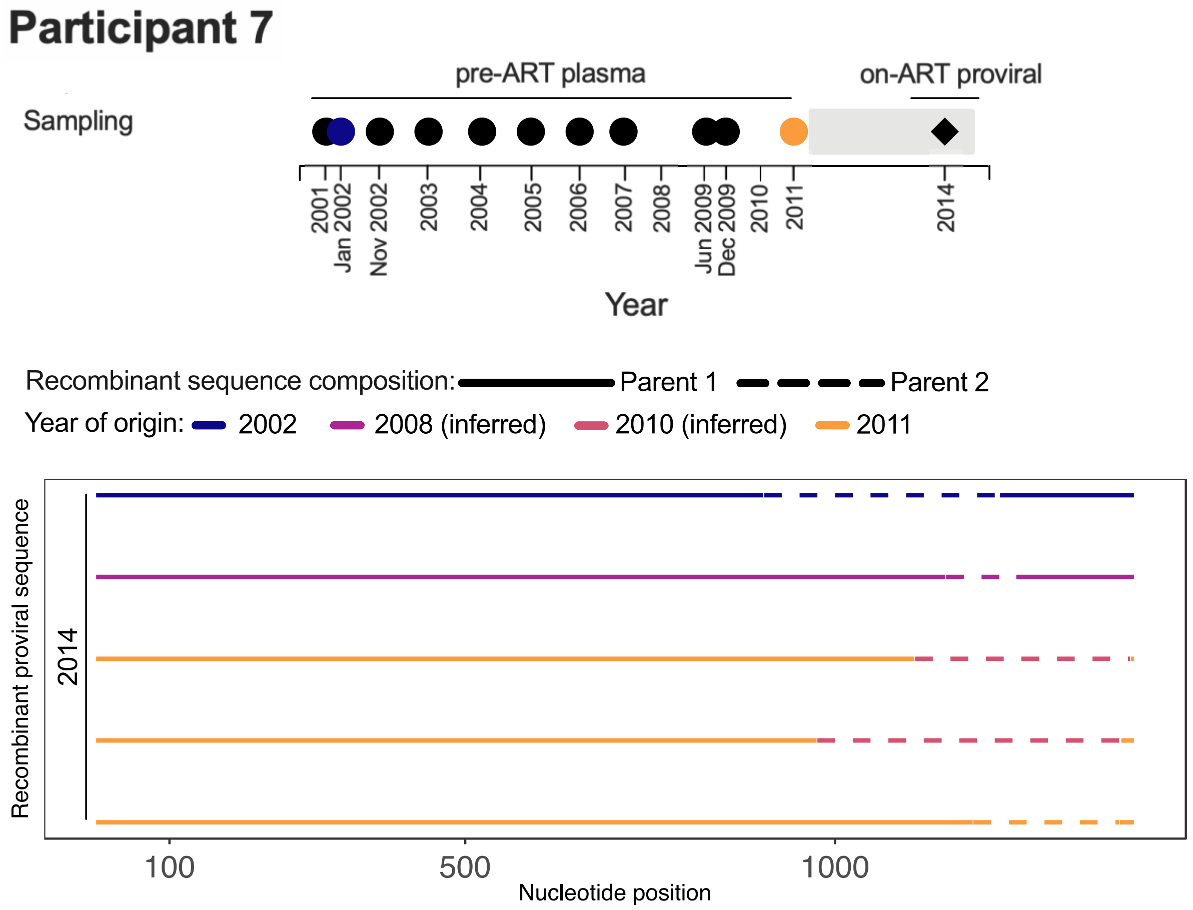

Fig. S6: Participant 7: recombinant *env-gp120* proviruses. Legend as in S3 Fig.

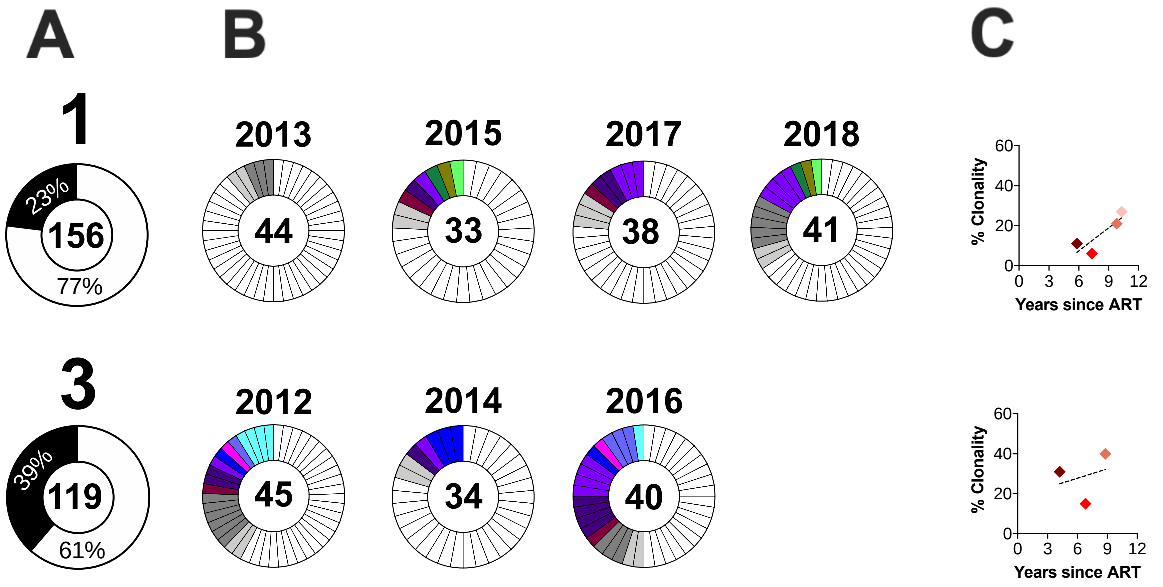

Fig. S7: Participants 1 and 3: *gag* proviral clonal distribution and dynamics during ART
(A) Total *gag* proviral sequences collected for participants 1 and 3 (shown inside the donut), and the percentage that were observed only once (white) those observed more than once (i.e., clones; black). (B) *gag* proviral clonality by time point. Grey slices denote clones distinct to that time point (each clone in a distinct shade of grey); colored slices link clones isolated across time points (C) Percent proviral clonality over time on ART, with regression line.

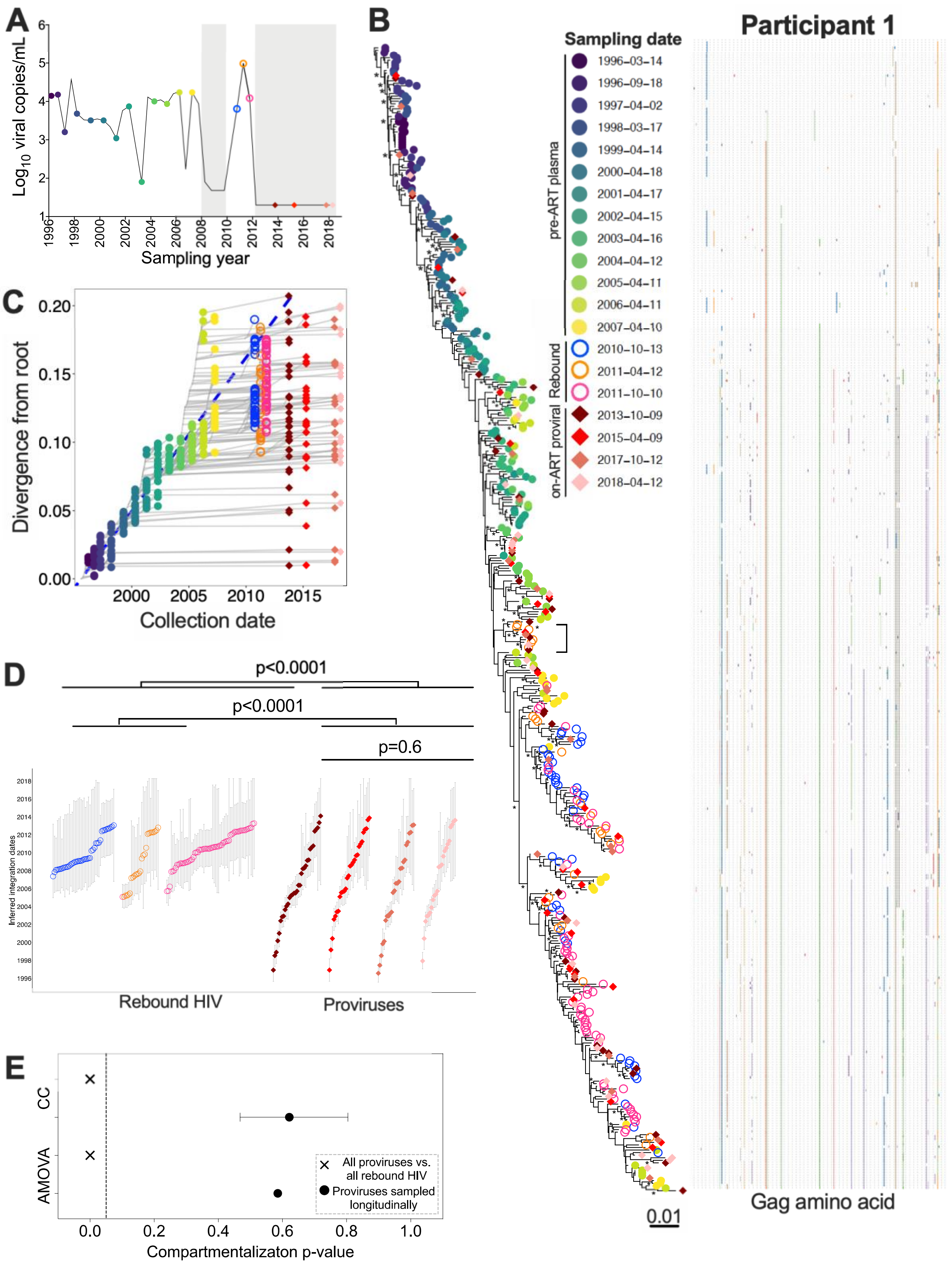

Fig. S8: Participant 1: diversity and inferred integration dates of HIV *gag* sequences persisting during ART. Legend as in Fig 3, except analysis is performed using *gag*.

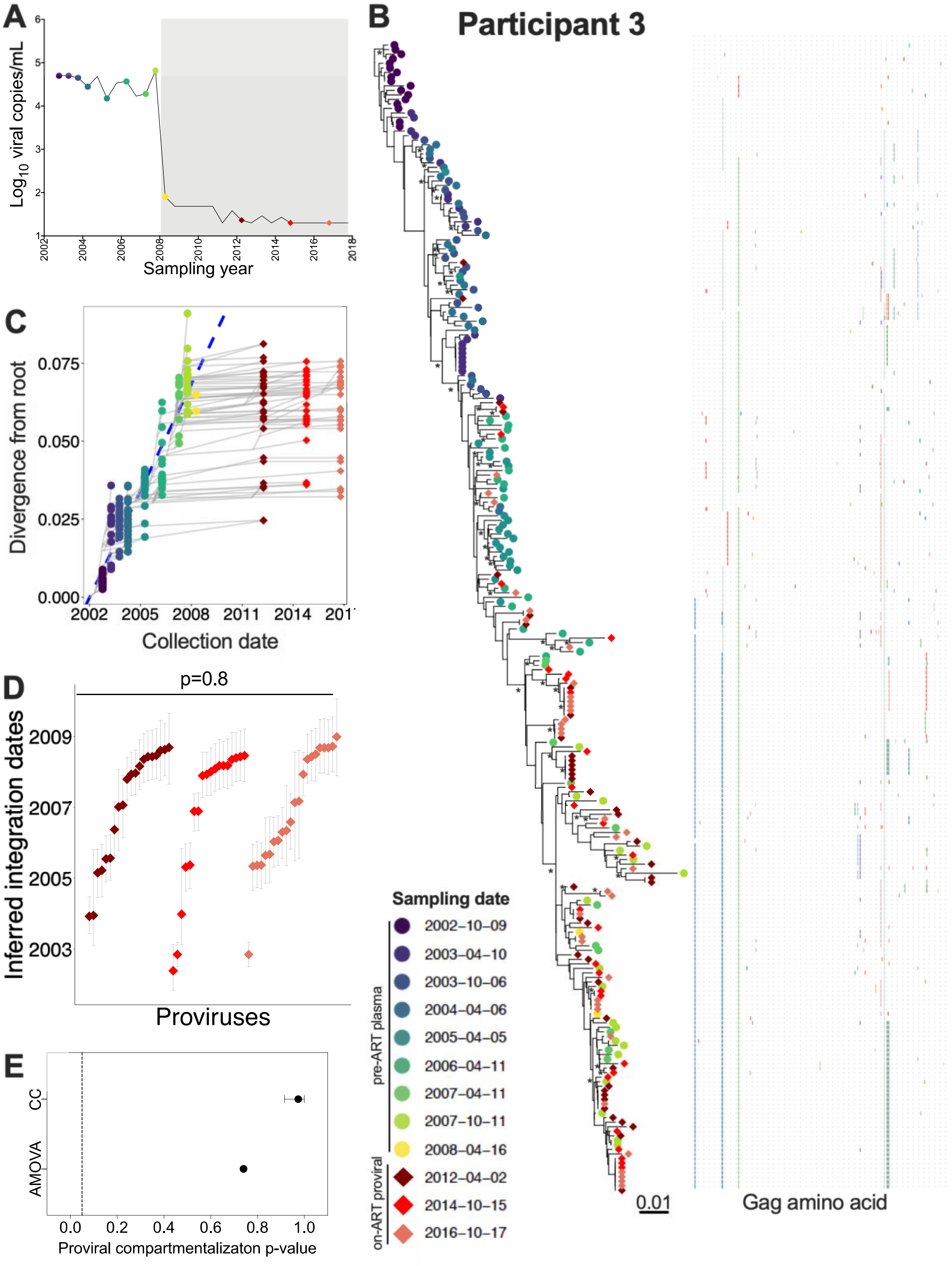

Fig. S9: Participant 3: diversity and inferred integration dates of HIV *gag* sequences persisting during ART. Legend as in Fig 6, except analysis is performed using *gag*.

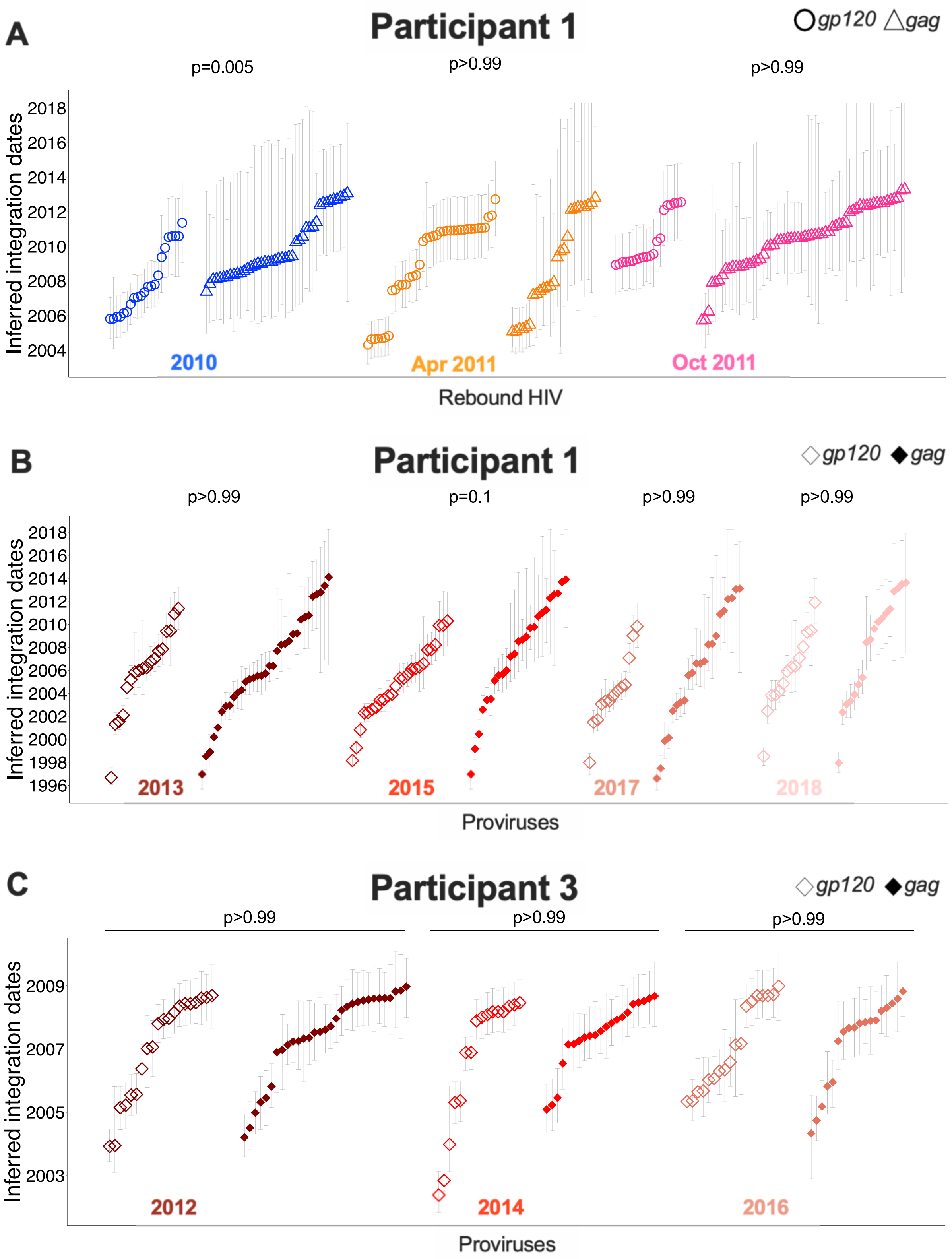

Fig. S10: Participants 1 and 3: comparison of *env-gp120* and *gag*-derived proviral integration dates. (A) Integration date estimate of each distinct rebound *env-gp120* (open circle) and *gag* (triangle) sequence recovered from participant 1, stratified by collection year. Whiskers denote 95% HPD intervals. (B) Integration date estimate of each distinct proviral *env-gp120* (open diamond) and *gag* (closed diamond) sequence recovered from participant 1, stratified by collection year. (C) same as (A), but for proviruses recovered from participant 3.

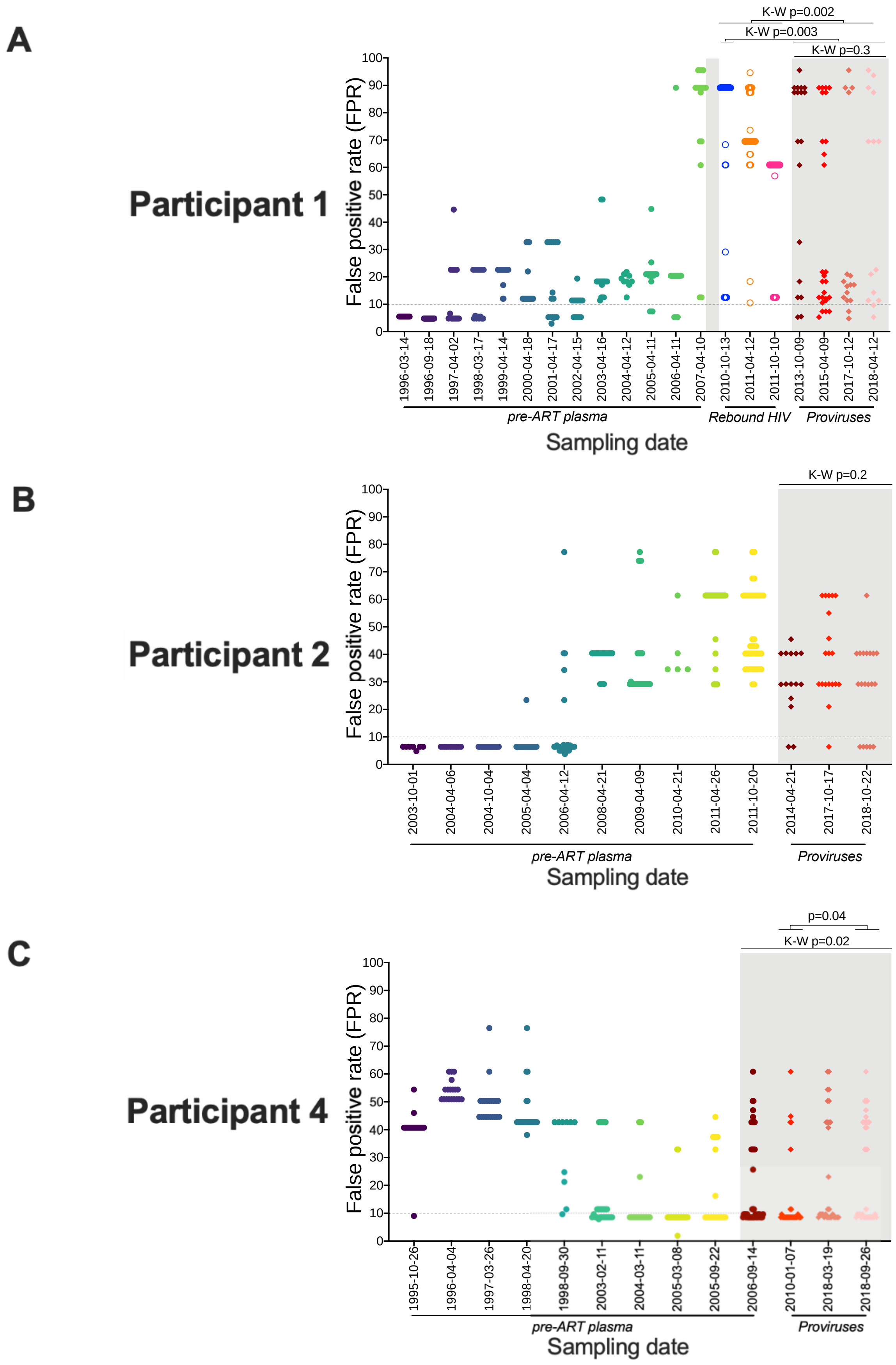

Fig. S11: Results validation using HIV co-receptor evolution. (A) The geno2pheno [coreceptor] false

positive rate (FPR) predictions for participant 1's distinct plasma HIV RNA (closed circles), rebound HIV (open circles) and proviral sequences (diamonds). Sequences with FPR ≤10% (dotted line) are considered X4. Grey shading denotes ART. Comparisons between FPR values across all longitudinal proviral on-ART time points is performed using a Kruskal-Wallis (K-W) test. Comparisons of FPR of proviral and rebound viruses are performed using the Mann-Whitney U-test. (B) same as (A) but for participant 2. (C) same as (A) but for participant 4. Here, p=0.02 is from the Kruskal-Wallis (K-W) test, while p=0.04 is the only statistically significant post-test, after correction for multiple comparisons.
