# Supplementary material for: The replication-competent HIV reservoir is a genetically restricted, younger subset of the overall pool of HIV proviruses persisting during therapy, which is highly genetically stable over time
Source: J Virol. 2024 Jan 12;98(2):e01655-23. doi: 10.1128/jvi.01655-23 (PMC10878278; doi:10.1128/jvi.01655-23)
Supplement: Table S1 — Bayesian within-host phylogenetic inference details. [file jvi.01655-23-s0002.pdf]

Table S1: **Bayesian within-host phylogenetic inference details**

Participant	MCMC generations[a]	Phylogenies post burn-in	QC-passed phylogenies	ΔAIC[b] (median, IQR)	Phylogenetically estimated root date (95% HPD[c])	Within-host HIV evolutionary rate[d] (mean, 95%HPD)
gp120						
1	100	15000	7218	488 (452 - 522)	Dec 1995 (Aug 1995 - Mar 1996)	4.8×10^{-5} (3.8×10^{-5} - 6.0×10^{-5})
2	35	5250	3842	588 (550 - 627)	May 2003 (Jan - Sep 2003)	5.9×10^{-5} (4.2×10^{-5} - $7.7\times10^{-5)}$
3	30	4500	4500	196 (181 - 213)	Nov 2001 (Jul 2001 - Mar 2002)	5.9×10^{-5} (4.2×10^{-5} - 7.6×10^{-5})
4	40	6000	6000	550 (525 - 574)	Feb 1995 (Oct 1994 - Jun 1995)	6.7×10^{-5} (5.5×10^{-5} - 8.1×10^{-5})
5	10	1500	1500	40 (34 - 48)	Dec 2008 (Oct 2008 - Feb 2009)	9.4×10^{-5} (5.3×10^{-5} - 1.4×10^{-4})
6	20	3000	2528	51 (45 - 60)	Jan 2007 (Sep 2006 - May 2007)	8.1×10^{-5} (6.1×10^{-5} - 1×10^{-4})
7	10	1500	1278	300 (273 - 328)	May 1999 (Jul 1998 - Dec 1999)	2.3×10^{-5} (1.7×10^{-5} - 2.9×10^{-5})
gag						
1	15	3000	2992	286 (254 - 317)	Feb 1995 (May - Nov 1995)	2.6×10^{-5} (1.9×10^{-5} - 3.2×10^{-5})
3	10	1500	1500	261 (239 - 283)	Nov 2001 (May 2001 - May 2002)	2.6×10^{-5} (2×10^{-5} - 3.5×10^{-5})
7	10	1500	1500	230 (209 - 254)	Feb 1998 (Dec 1996 - Apr 1999)	1.3×10^{-5} (9.0×10^{-5} - 1.6×10^{-5})

[a] jModelTest returned GTR+I+G (General Time Reversible model with Invariable site plus Gamma distribution with four rate categories) as the best-fit nucleotide substitution model for all participants' *gp120* and *gag* nucleotide sequence alignments.

[b] ΔAIC, delta Akaike information criterion. A phylogeny required a ΔAIC of ≥ 10 (where its root date was prior to the first plasma sampling) was considered evidence of a molecular clock signal (see methods).

[c] 95% HPD, 95% highest posterior density.

[d] Expressed in estimated substitutions per nucleotide site per day.
